# Effect of dietary habits on multiple cardiovascular diseases: A comprehensive Mendelian randomization study

**DOI:** 10.1097/MD.0000000000044352

**Published:** 2025-09-05

**Authors:** Youfu He, Qiang Wu, Debin Liu, Xuantong Meng, Yu Qian

**Affiliations:** a Department of Cardiology, Guizhou Provincial People’s Hospital, Guiyang, Guizhou Province, China; b Department of Cardiology, Affiliated People’s Hospital of Guizhou University, Guiyang, Guizhou Province, China; c Department of Cardiology, The Second People’s Hospital of Shantou, Shantou, Guangdong Province, China; d Department of Pathology, 79th Army Group Military Hospital, Liaoyang, Liaoning Province, China; e Department of Cardiology, The Second Affiliated Hospital of Zunyi Medical University, Zunyi, Guizhou Province, China.

**Keywords:** bioinformatics analysis, cardiovascular disease, co-location analysis, dietary habits, meta-analysis, two-sample Mendelian randomization

## Abstract

The relationship between dietary habits, including the consumption of eggs, dairy products, and sugar, and the development of disease is well-established. However, further investigation is required to elucidate the precise associations between these dietary habits and cardiovascular disease (CVD). The objective of this study was to analyze existing genome-wide association studies in order to identify causal relationships between dietary habits and CVD. The 5 dietary habits rooted from the IEU database are categorized as: *Never eat eggs*, *Never eat wheat products*, *Never eat sugar or foods/drinks containing sugar*, *Never eat wheat products*, and *I eat all of the above*. CVDs are categorized into: *heart failure, Myocardial infarction(MI), Arrhythmia, Hypertension, Coronary heart disease(CHD), Myocarditis, Cardiomyopathy, Valvular heart disease*, and *cardiac death*. This was achieved by employing two-sample Mendelian randomization and co-localization analyses. The potential causative genes associated with 5 dietary habits and CVDs were also explored through bioinformatics and receiver operating characteristic curve analysis based on GeneChip GSE59867. Subsequently, we employed meta-analysis techniques to integrate the findings of these studies. The co-localization analysis, based on two-sample Mendelian randomization results, revealed that single nucleotide polymorphism (SNP) rs12740374 (PH4 = 0.855) was causally associated with MI and a balanced diet(I eat all of the above), while SNP rs7528419 (PH4 = 0.716) was causally associated with CHD and a balanced diet. Moreover, 2 SNPs, rs7528419 and rs12740374, were identified as drivers of common motifs in 6 genes. The following genes were identified as having driver effects on common motifs: KIAA1324, SARS, CELSR2, PSRC1, MYBPHL, and SORT1. PSRC1 (area under the curve [AUC]: 0.722), CELSR2 (AUC: 0.657), and MYBPHL (AUC: 0.677) have been identified as having high diagnostic value for CHD and MI. The results of the study indicate a strong correlation between dietary habits and a multitude of CVDs. In almost all populations, diets that do not consume eggs, dairy products, and sugar is a significant risk factor for CVD in nearly all groups, whereas a balanced diet is a protective factor for the majority of CVDs.

## 1. Introduction

Cardiovascular disease (CVD) is the leading cause of death globally.^[1]^ The International Guidelines for the Prevention of CVD indicate that unhealthy eating habits and poor lifestyles are associated with CVD development. The optimization of dietary structure stands as a pivotal strategy in reducing CVD risk.^[2]^ Conversely, inappropriate dietary patterns can be a crucial factor in the onset of CVD.^[3]^ Studies confirm that over 50% of CVD-related deaths and disabilities are attributable to dietary risk factors.^[4]^ Consequently, developing a healthy dietary pattern for the primary prevention of CVD is a pressing research topic in the field of CVD prevention and treatment.

Balanced nutrition is crucial for maintaining human health.^[5]^ However, the impact of dairy products such as butter and cream, carbohydrates like sugar and wheat products, as well as eggs on CVD have been the topic of debate in several prior studies. Certain studies have indicated that diets high in carbohydrates like sugar and wheat may increase the risk of heart damage, CVD, and hypertension.^[6,7]^ In contrast, some experts have recommended a high-carbohydrate, low-fat diet for individuals with heart failure (HF) or hypertension due to the fact that a high-carbohydrate diet activates the PPAR system, which promotes the expression of protective cardiac genes and cell growth.^[8]^ On the other hand, eggs and dairy products such as butter and cream have been criticized for their high cholesterol and saturated fat content.^[9]^ One study found a significant correlation between egg intake and CVD events and all-cause mortality among adults in the United States.^[10]^ However, another study has documented a significant negative association between egg consumption and coronary heart disease (CHD; pooled hazard ratios, 0.89; 95% confidence interval, 0.86–0.93; *P* < .001; *I*² = 0%).^[11]^ Furthermore, studies have demonstrated that the intake of butter, which is abundant in saturated fatty acids, significantly increases the concentration of LDL-C in humans.^[12]^ There is also a strong positive correlation between elevated LDL-C levels and CVD.^[13]^ However, recent research suggests that consuming dairy products is associated with lower mortality rates (RR 0.98, 95% CI 0.97–0.99; *I*^2^ = 94.4%) as well as reduced risk of CVD (RR 0.98, 95% CI 0.97–0.99; *I*^2^ = 87.5%).^[14]^ So the association between different dietary structures and CVDs is actually not very clear and still deserves further exploration by researchers.

To explore the correlation between dairy products, carbohydrates (sugary and wheat-based foods), eggs, and CVDs, we leveraged the respective single nucleotide polymorphisms (SNPs) sourced from the publicly accessible genome-wide association studies (GWAS) database. Employing Mendelian randomization (MR) analysis and meta-analysis, we conducted comprehensive assessments. Our objective was to reveal the impact of dietary patterns on various CVDs from a genomic standpoint.

## 2. Methods

### 2.1. Design of experiments

In this study, two-sample Mendelian randomization (TSMR) coupled with expression quantitative trait loci-based co-localization analysis was employed to investigate potential causal links between CVD and dietary habits. Dietary habits were defined as “Never eat eggs or foods containing eggs,” “Never eat dairy products,” “Never eat wheat products,” “Never eat sugar or foods/drinks containing sugar,” and “having a balanced diet (I eat all of the above).” CVD was defined as all-cause HF; myocardial infarction (MI); arrhythmia; essential hypertension; CHD; myocarditis; cardiomyopathy; valvular disease; and cardiac death. This study proceeded in 3 steps. Step 1 involved sequential TSMR analyses, utilizing 5 dietary habits as exposure variables and various CVDs as outcomes. Subsequently, Step 2 entailed a meta-analysis aggregating the TSMR outcomes across groups. Finally, in Step 3, a co-localization analysis sought potential target genes for disease treatment.

### 2.2. GWAS data sources

The study leveraged data from 4 publicly available databases: UK Biobank (www.ukbiobank.ac.uk/), IEU (https://gwas.mrcieu.ac.uk/), FinnGen (https://www.finngen.fi/en), and the GWAS catalog (https://www.ebi.ac.uk/gwas/). The 5 diets were obtained from UK Biobank, with a cumulative sample of 23,05,230 cases. All studies were based on samples of Europeans.

The study’s inclusion criteria were as follows: The study had to be from a different database source, with at least 3 studies included for each disease. If a relevant study was not found in a particular database, the most recent study from the remaining databases was selected as a supplement. The included studies had to have sample sizes of more than 1,00,000. If the study was present in fewer than three of the 4 available databases, it was included in all of them.

CVD was defined as follows: All cases of HF, irrespective of cause or severity, were included in a total of 3 studies from the GWAS catalog and Finngen, with a total sample size of 9,12,546 cases. Additionally, cases of MI, defined as any instance of MI regardless of cause or type, were enrolled in a total of 4 studies from IEU, GWAS catalog, and Finngen, with a total sample size of 1,53,056 cases. Arrhythmia, defined as any type of irregular heartbeat not specific to its cause, was included in 3 studies from the IEU, GWAS catalog, and UK Biobank, with a combined sample size of 11,59,984 cases. Essential hypertension, defined as high blood pressure without distinguishing between grade and risk, was enrolled in 3 studies from the GWAS catalog and UK Biobank included a total sample size of 14,10,541 cases. Essential hypertension (defined as essential hypertension without differentiation of grade and risk) was enrolled in a total of 3 studies from the GWAS catalog and UK Biobank, with a total sample size of 14,10,541 cases. Enrollment for CHD (defined as CHD regardless of cause and degree) involved a total of 3 studies from the GWAS catalog and IEU, with a sample size of 10,83,629 cases. Enrollment for myocarditis (defined as myocarditis regardless of cause) involved a total of 3 studies from the GWAS catalog and Finngen, with a sample size of 7,13,513 cases. Cardiomyopathy, defined as a condition of the heart muscle without distinguishing the cause, was studied in 3 different research projects, all from Finngen, with a combined sample size of 5,35,590 cases. Valvulopathy, defined as valvulopathy without differentiation of cause, was studied in a total of 3 studies conducted by the IEU, UK Biobank, and Finngen, totaling 11,42,451 cases. Cardiac death, defined as death resulting from CVD, was studied in a total of 2 studies conducted by UK Biobank and Finngen, with an overall sample size of 5,80,186 cases.

Additional details including all GWAS-IDs and data sources can be found in Table S1, Supplemental Digital Content, https://links.lww.com/MD/P887.

### 2.3. Instrumental variables and data harmonization options

MR serves as a method for exploring causality utilizing instrumental variables (IVs), where SNPs commonly serve as genetic IVs. MR designs must follow 3 assumptions: genetic IVs exhibit high linkage to the exposure; genetic IVs remain distinct from potential confounders; and genetic IVs exclusively impact outcomes through risk factors. Our approach encompassed various techniques to select validated IVs meeting these assumptions. Firstly, SNPs demonstrating a significant association with the exposure were chosen, employing a stringent threshold of *P* < 5 × 10^−6^ due to SNP availability constraints. Subsequently, SNPs exhibiting strong linkage disequilibrium (LD *R*^2^ < 0.001, LD distance > 10,000 kb) were excluded. Lastly, potential SNPs displaying multiple associations were identified through the PhenoScanner V2 database, with SNPs linked to potential confounders eliminated from the analysis. Additionally, SNPs demonstrating inconsistent allele orientations between exposure and outcome were excluded to mitigate the possibility of reverse causation.

### 2.4. Sensitivity analyses

A sensitivity analysis was conducted using the leave-one-out analysis to examine the effect of each SNP on the results 1 by 1.

### 2.5. Statistics and analyses

The statistical analyses were performed utilizing R software (v4.3.0) along with the Two-Sample MR (version 0.5.6) and Colo (version 2.0) packages. The TSMR analysis involved a combination of random effects inverse variance weighted (IVW), weighted median, simple mode, MR-Egger, weighted mode. The IVW method produced the most significant findings with statistical significance set at *P* < .05. Co-localization analysis was performed by generating ±500 kb windows around the gene encoding each respective drug target. This analysis utilized approximate Bayesian factorials, which provided posterior probabilities (PP) representing the associations between 2 traits in various configurations: neither trait demonstrated genetic association in the region (PP.H0); only the first trait exhibited genetic association in the region (PP.H1); only the second trait demonstrated genetic association in the region (PP.H2); both traits were associated but with different causal variants (H3); and both traits were associated and shared a causal variant (PP.H4).^[15]^ We employed a threshold of PP.H4 > 0.7 to denote substantial support for the tested configuration.^[16]^

We obtained the peripheral-blood whole-genome microarray dataset associated with MI from the GEO database (https://www.ncbi.nlm.nih.gov/GEO/) to evaluate the predictive potential of candidate genes. The dataset GSE59867 comprises peripheral-blood samples collected from 111 patients previously diagnosed with ST-segment elevation myocardial infarction (STEMI) and 46 stable CAD patients without a history of MI. The “pROC” package facilitated the computation of receiver operating characteristic (ROC) curves, area under the curve (AUC) values, and corresponding 95% confidence intervals (95% CI). An AUC > 0.6 indicated high accuracy or sensitivity.^[17]^

## 3. Results

### 3.1. Research design

**Figure 1.**
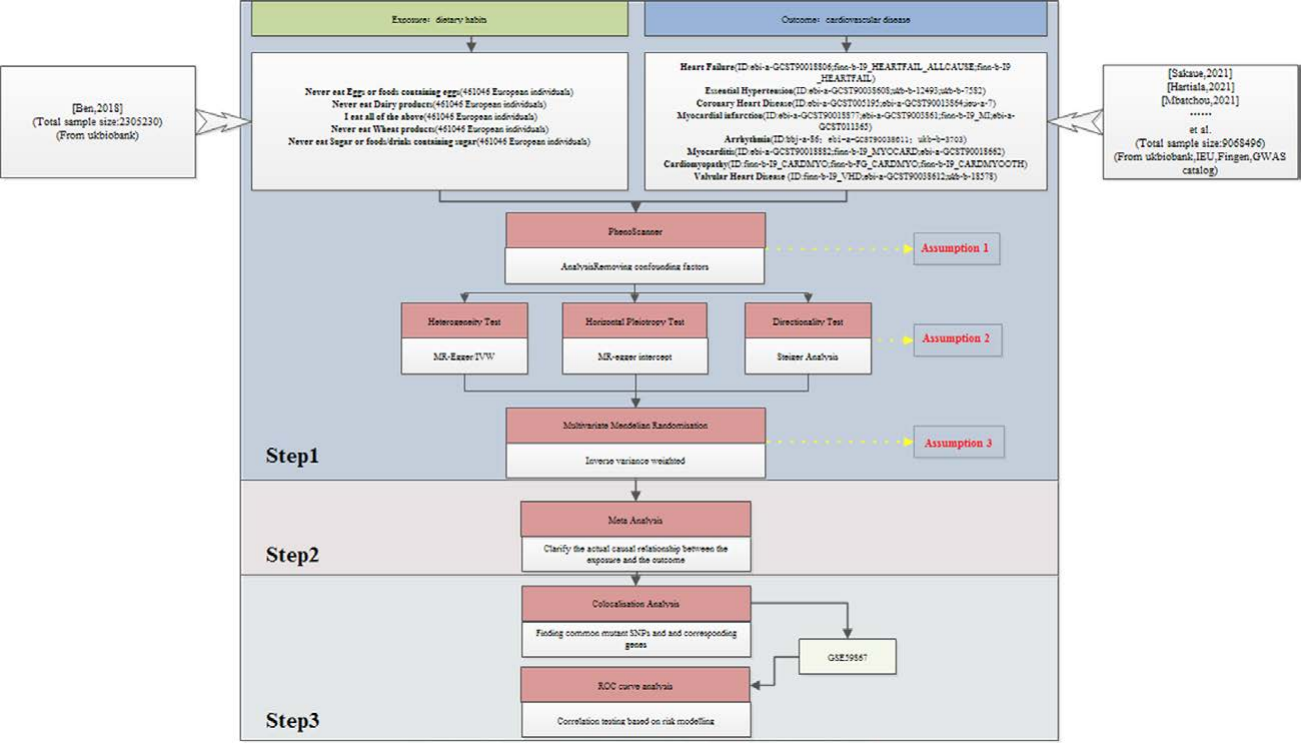
Mendelian randomization study design. GWAS = genome-wide association studies, IEU = integrative epidemiology unit, IVW = inverse variance weighted, MR = Mendelian randomization, ROC = receiver operating characteristic, SNP = single nucleotide polymorphism.

The study’s design, as shown in Figure 1, follows the MR method. The instrumental variable SNPs were screened based on the 3 MR assumptions: correlation (Assumption 1), independence (Assumption 2), and exclusivity (Assumption 3). The study used the TSMR approach, which consists of 3 steps.

Step 1: Sequential TSMR analyses were performed, utilizing 5 dietary habits as exposure variables and various CVDs as outcome variables.

Step 2: A meta-analysis was conducted, categorizing various CVDs and integrating the TSMR results within each group.

Step 3: Positive findings from the meta-analysis underwent co-localization analysis to identify common driver SNPs and their respective genes. Following the co-localization analysis results, appropriate GEO chips were selected for ROC analyses to assess the susceptibility of chosen genes to the disease.

### 3.2. Two-sample Mendelian randomization analysis

#### 3.2.1. Heterogeneity, pleiotropy, and directionality testing

At the study’s outset, PhenoScanner was employed to initially eliminate confounding variables. Table S2, Supplemental Digital Content, https://links.lww.com/MD/P887 presents the primary findings related to heterogeneity, pleiotropy, and directionality within the study, together with the number of SNPs included in the study. The MR-Egger intercept values consistently approximated 0 across all TSMR analyses, yielding MR-Egger intercept *P*-values exceeding .05, suggesting the absence of horizontal pleiotropy among the study variables. Cochran’s *Q*-test was used to detect heterogeneity, with a *Q*-*p*val > 0.05 indicating no evidence of heterogeneity. Consequently, we opted for estimating the MR effect size using the IVW fixed-effects model. In scenarios where MR-Egger or IVW *P* < .05, indicating potential heterogeneity among study variables, we transitioned to utilizing the IVW random effects model for estimating the MR effect size. All TSMR analyses passed the Steiger direction test in this study, and the results indicated no reverse causality as the Steiger analysis results were all TRUE. The leave-one-out analysis test did not identify any significant SNPs influencing the estimates of causal association, underscoring the robustness of our findings. The funnel plots depicting the causal effect distribution exhibited basic symmetry and indicated no discernible bias (please refer to Figs. S1–S9, Supplemental Digital Content, https://links.lww.com/MD/P886 for leave-one-out analysis plots and funnel plots post confounder removal).

#### 3.2.2. TSMR results

The TSMR analysis revealed a positive association between “Never Eat Eggs” and cardiac death (exposure-ID: ukb-d-I9_K_CARDIAC). A 1 standard deviation increase in “Never eat eggs” exposure escalated the risk of cardiac death (OR = 1.116; 95% CI = 1.038–1.201; FDR-*P* = .017). Conversely, “Never eat dairy products” exhibited no evident cause-effect relationship with several discussed CVDs (FDR-*P* > .1). However, “Never eat wheat products” showed a positive association with essential hypertension (exposure-ID: ukb-b-7582). Each standard deviation increase in “Never eat wheat products’ elevated the risk of essential hypertension (OR = 1.037; 95% CI = 1.007–1.067; FDR-*P* = .08). Moreover, “Never eat sugar or sugary foods” demonstrated positive associations with HF (exposure IDs: ebi-a-GCST90018806, finn-b-I9_HEARTFAIL_ALLCAUSE), essential hypertension (exposure IDs: ukb-b-12493, ukb-b-7582), and CHD (exposure ID: ebi-a-GCST90013864). The risk correlations ORs and *P*-values showed slight variations depending on the GWAS ID of the studies included. Each 1 standard deviation increase in “Never eat sugar” corresponded to an increased risk of HF (ebi-a-GCST90018806: OR = 2.804, 95% CI = 1.206–6.519, FDR-*P* = .051; finn-b-I9_HEARTFAIL_ALLCAUSE: OR = 4.020, 95% CI = 1.451–11.139, FDR-*P* *=* .026), essential hypertension (ukb-b-12493: OR = 1.148, 95% CI = 1.084–1.216, FDR-*P* < .001). Interestingly, a “balanced diet” (I eat all of the above) displayed significant negative correlations (FDR-*P* < .1) with HF (exposure IDs: ebi-a-GCST90018806, Finn-b-I9_HEARTFAIL_ALLCAUSE, Finn-b-I9_HEARTFAIL), essential hypertension (ukb-b-12493), CHD (IDs: ebi-a-GCST005195, ebi-a-GCST90013864, ieu-a-7), MI (ebi-a-GCST90018877, Finn-b-I9_MI, ebi-a-GCST011365, ebi-a-GCST90038610), valvular disease (finn-b I9_VHD), and cardiac death (finn-b-I9_K_CARDIAC, ukb-d-I9_K_CARDIAC).

Specific results are presented in Table 1, while Figure S10, Supplemental Digital Content, https://links.lww.com/MD/P886 displays forest plots and scatter plots of the conventional 5 analyses (IVW, MR-Egger, simple mode, weighted median, and weighted mode). The findings indicate that the results of the conventional 5 analyses for each group of analyses are consistent, implying strong reliability and robustness of the results.

**Table 1:** Two-sample Mendelian randomization analysis results.

Disease	Outcome-name	Outcome-ID	Exposure-name	Exposure-ID	MR-Egger				Inverse variance weighted					Weighted mode			
					*P*	or	or_lci95	or_uci95	*P*	FDR-*P*	or	or_lci95	or_uci95	*P*	or	or_lci95	or_uci95
Heart failure	Chronic heart failure	ebi-a-GCST90018806	Never eat eggs, dairy, wheat, sugar: eggs or foods containing eggs	ukb-b-17455	.603	0.001	0.000	1.44E+07	.529	.721364	6.213	0.021	1.82E+03	.338	3.75E+02	0.00	2.62E+07
			Never eat eggs, dairy, wheat, sugar: dairy products	ukb-b-18909	.674	491.774	0.000	3.10E+13	.120	.257143	224.401	0.243	2.07E+05	.674	12.803	0.000	6.04E+05
			Never eat eggs, dairy, wheat, sugar: I eat all of the above	ukb-b-2393	.143	0.096	0.004	2.124	.000	0	0.231	0.118	0.451	.035	0.134	0.021	0.833
			Never eat eggs, dairy, wheat, sugar: wheat products	ukb-b-3599	.309	0.251	0.022	2.875	.147	.273333	0.271	0.046	1.582	.149	0.175	0.021	1.437
			Never eat eggs, dairy, wheat, sugar: sugar or foods/drinks containing sugar	ukb-b-5495	.780	0.489	0.003	71.324	.017	.051	2.804	1.206	6.519	.228	3.594	0.460	28.099
	All-cause heart failure	finn-b-I9_HEARTFAIL_ALLCAUSE	Never eat eggs, dairy, wheat, sugar: eggs or foods containing eggs	ukb-b-17455	.676	1.02E+03	0.000	1.31E+16	.857	.877	2.205	0.000	1.21E+04	.454	0.018	0.000	2.84E+02
			Never eat eggs, dairy, wheat, sugar: dairy products	ukb-b-18909	.663	3.08E+03	0.000	1.04E+17	.790	.877	3.254	0.001	1.94E+04	.616	33.891	0.000	8.07E+06
			Never eat eggs, dairy, wheat, sugar: I eat all of the above	ukb-b-2393	.743	0.592	0.026	13.468	.000	0	0.200	0.100	0.401	.190	0.237	0.028	1.993
			Never eat eggs, dairy, wheat, sugar: wheat products	ukb-b-3599	.088	105.716	1.817	6.15E+03	.296	.444	5.645	0.219	145.401	.103	25.745	1.059	625.941
			Never eat eggs, dairy, wheat, sugar: sugar or foods/drinks containing sugar	ukb-b-5495	.508	8.016	0.018	3.63E+03	.007	.02625	4.020	1.451	11.139	.193	3.948	0.512	30.424
	Heart failure, strict	finn-b-I9_HEARTFAIL	Never eat eggs, dairy, wheat, sugar: eggs or foods containing eggs	ukb-b-17455	.702	8.00E+03	0.000	3.05E+22	.624	.78	20.281	0.000	3.46E+06	.387	0.004	0.000	3.98E+02
			Never eat eggs, dairy, wheat, sugar: dairy products	ukb-b-18909	.706	7.66E+02	0.000	7.33E+15	.164	.273333	340.804	0.093	1.25E+06	.752	9.099	0.000	2.38E+06
			Never eat eggs, dairy, wheat, sugar: I eat all of the above	ukb-b-2393	.724	0.511	0.013	20.664	.000	0	0.142	0.062	0.324	.089	0.082	0.005	1.405
			Never eat eggs, dairy, wheat, sugar: wheat products	ukb-b-3599	.354	16.383	0.087	3.08E+03	.877	.877	1.323	0.038	46.519	.440	5.641	0.098	323.099
			Never eat eggs, dairy, wheat, sugar: sugar or foods/drinks containing sugar	ukb-b-5495	.937	1.302	0.002	8.41E+02	.068	.17	2.727	0.927	8.021	.829	1.360	0.084	21.929
Essential hypertension	Diagnoses – secondary ICD10: I10 essential (primary) hypertension	ukb-b-12493	Never eat eggs, dairy, wheat, sugar: eggs or foods containing eggs	ukb-b-17455	.933	1.120	0.090	1.40E+01	.555	.756818	0.829	0.444	1.546	.955	0.981	0.519	1.854
			Never eat eggs, dairy, wheat, sugar: dairy products	ukb-b-18909	.789	0.781	0.160	3.80E+00	.201	.376875	0.753	0.487	1.164	.842	0.926	0.461	1.859
			Never eat eggs, dairy, wheat, sugar: I eat all of the above	ukb-b-2393	.227	0.900	0.759	1.066	.000	0	0.842	0.801	0.884	.035	0.859	0.748	0.987
			Never eat eggs, dairy, wheat, sugar: wheat products	ukb-b-3599	.660	0.964	0.824	1.128	.153	.362143	1.077	0.973	1.194	.772	1.021	0.893	1.167
			Never eat eggs, dairy, wheat, sugar: sugar or foods/drinks containing sugar	ukb-b-5495	.350	1.108	0.895	1.371	.000	0	1.148	1.084	1.216	.078	1.132	0.989	1.297
	Non-cancer illness code, self-reported: essential hypertension	ukb-b-7582	Never eat eggs, dairy, wheat, sugar: eggs or foods containing eggs	ukb-b-17455	.382	0.829	0.553	1.244	.696	.803077	1.010	0.960	1.064	.594	0.967	0.858	1.090
			Never eat eggs, dairy, wheat, sugar: dairy products	ukb-b-18909	.718	0.840	0.336	2.099	.689	.803077	1.016	0.940	1.098	.346	1.083	0.925	1.268
			Never eat eggs, dairy, wheat, sugar: I eat all of the above	ukb-b-2393	.477	1.018	0.969	1.071	.169	.362143	0.995	0.988	1.002	.925	1.001	0.977	1.026
			Never eat eggs, dairy, wheat, sugar: wheat products	ukb-b-3599	.188	1.038	0.984	1.096	.016	.08	1.037	1.007	1.067	.049	1.048	1.003	1.094
			Never eat eggs, dairy, wheat, sugar: sugar or foods/drinks containing sugar	ukb-b-5495	.087	1.065	0.992	1.143	.036	.135	1.010	1.001	1.020	.313	1.014	0.987	1.042
	Essential hypertension	ebi-a-GCST90038608	Never eat eggs, dairy, wheat, sugar: eggs or foods containing eggs	ukb-b-17455	.603	0.933	0.731	1.191	.381	.5715	0.971	0.910	1.037	.435	0.948	0.838	1.074
			Never eat eggs, dairy, wheat, sugar: dairy products	ukb-b-18909	.978	1.007	0.661	1.533	.914	.979286	0.995	0.906	1.093	.798	1.021	0.880	1.185
			Never eat eggs, dairy, wheat, sugar: I eat all of the above	ukb-b-2393	.642	1.007	0.978	1.037	.999	.999	1.000	0.993	1.007	.939	1.001	0.977	1.026
			Never eat eggs, dairy, wheat, sugar: wheat products	ukb-b-3599	.701	0.992	0.957	1.029	.380	.5715	1.010	0.987	1.034	.575	0.99	0.963	1.021
			Never eat eggs, dairy, wheat, sugar: sugar or foods/drinks containing sugar	ukb-b-5495	.488	1.021	0.963	1.084	.119	.357	1.008	0.998	1.017	.323	1.014	0.987	1.042
Coronary heart disease	Coronary artery disease	ebi-a-GCST005195	Never eat eggs, dairy, wheat, sugar: eggs or foods containing eggs	ukb-b-17455	.519	9.41E+03	0.000	1.00E+15	.640	.872727	0.194	0.000	187.118	.526	10.846	0.011	1.04E+04
			Never eat eggs, dairy, wheat, sugar: dairy products	ukb-b-18909	.850	0.158	0.000	3.41E+06	.129	.302143	0.028	0.000	2.839	.676	0.170	0.000	3.21E+02
			Never eat eggs, dairy, wheat, sugar: I eat all of the above	ukb-b-2393	.044	0.060	0.004	0.865	.000	0	0.199	0.099	0.397	.084	0.185	0.028	1.209
			Never eat eggs, dairy, wheat, sugar: wheat products	ukb-b-3599	.168	0.179	0.024	1.334	.141	.302143	0.342	0.082	1.424	.127	0.250	0.057	1.107
			Never eat eggs, dairy, wheat, sugar: sugar or foods/drinks containing sugar	ukb-b-5495	.063	22.364	0.922	5.43E+02	.139	.302143	1.512	0.874	2.615	.150	3.712	0.642	21.451
	Coronary artery disease (Firth correction)	ebi-a-GCST90013864	Never eat eggs, dairy, wheat, sugar: eggs or foods containing eggs	ukb-b-17455	.692	1.36E+03	0.000	3.34E+17	.435	.6525	0.030	0.000	197.618	.890	0.471	0.000	1.16E+04
			Never eat eggs, dairy, wheat, sugar: dairy products	ukb-b-18909	.798	24.439	0.000	5.29E+10	.384	.64	0.072	0.000	26.729	.936	0.650	0.000	9.92E+03
			Never eat eggs, dairy, wheat, sugar: I eat all of the above	ukb-b-2393	.031	0.024	0.001	0.652	.000	0	0.135	0.057	0.322	.014	0.045	0.004	0.497
			Never eat eggs, dairy, wheat, sugar: wheat products	ukb-b-3599	.754	0.702	0.083	5.900	.823	.877	0.853	0.212	3.427	.829	0.822	0.147	4.594
			Never eat eggs, dairy, wheat, sugar: sugar or foods/drinks containing sugar	ukb-b-5495	.125	27.605	0.433	1.76E+03	.030	.1125	2.168	1.079	4.356	.808	1.301	0.158	10.681
	Coronary heart disease	ieu-a-7	Never eat eggs, dairy, wheat, sugar: eggs or foods containing eggs	ukb-b-17455	.752	44.172	0.000	1.96E+11	.720	.877	0.387	0.002	68.888	.445	0.020	0.000	2.38E+02
			Never eat eggs, dairy, wheat, sugar: dairy products	ukb-b-18909	.669	4.00E+03	0.000	6.54E+17	.831	.877	0.459	0.000	579.984	.824	0.298	0.000	5.39E+03
			Never eat eggs, dairy, wheat, sugar: I eat all of the above	ukb-b-2393	.541	0.435	0.031	6.184	.000	0	0.246	0.132	0.461	.487	0.476	0.060	3.804
			Never eat eggs, dairy, wheat, sugar: wheat products	ukb-b-3599	.740	0.513	0.013	20.454	.877	.877	0.813	0.059	11.270	.967	0.938	0.052	16.816
			Never eat eggs, dairy, wheat, sugar: sugar or foods/drinks containing sugar	ukb-b-5495	.859	1.507	0.017	133.911	.201	.376875	1.620	0.773	3.394	.623	1.616	0.241	10.814
Myocardial infarction	Myocardial infarction	ebi-a-GCST90018877	Never eat eggs, dairy, wheat, sugar: eggs or foods containing eggs	ukb-b-17455	.683	2.22E+03	0.000	3.19E+18	.992	.992	1.045	0.000	3.96E+03	.410	46.629	0.009	2.29E+05
			Never eat eggs, dairy, wheat, sugar: drairy products	ukb-b-18909	.871	8.549	0.000	7.90E+10	.265	.448333	0.031	0.000	14.062	.349	0.006	0.000	51.214
			Never eat eggs, dairy, wheat, sugar: I eat all of the above	ukb-b-2393	.055	0.048	0.002	1.002	.000	0	0.195	0.098	0.386	.059	0.150	0.022	1.035
			Never eat eggs, dairy, wheat, sugar: wheat products	ukb-b-3599	.171	0.092	0.005	1.864	.100	.333333	0.158	0.018	1.425	.075	0.123	0.017	0.879
			Never eat eggs, dairy, wheat, sugar: sugar or foods/drinks containing sugar	ukb-b-5495	.555	4.861	0.026	893.256	.379	.583077	1.482	0.617	3.562	.076	5.130	0.872	30.178
	Myocardial infarction	finn-b-I9_MI	Never eat eggs, dairy, wheat, sugar: eggs or foods containing eggs	ukb-b-17455	.740	152.022	0.000	1.63E+14	.948	.992	1.307	0.000	3.89E+03	.956	0.633	0.000	3.37E+06
			Never eat eggs, dairy, wheat, sugar: dairy products	ukb-b-18909	.834	27.254	0.000	1.94E+13	.431	.615714	0.028	0.000	2.04E+02	.450	0.004	0.000	1.14E+03
			Never eat eggs, dairy, wheat, sugar: I eat all of the above	ukb-b-2393	.613	3.784	0.022	643.940	.006	.03	0.199	0.063	0.628	.667	2.525	0.038	167.311
			Never eat eggs, dairy, wheat, sugar: wheat products	ukb-b-3599	.069	1.32E+03	4.450	3.91E+05	.145	.3625	19.950	0.355	1.12E+03	.086	109.825	1.471	8.20E+03
			Never eat eggs, dairy, wheat, sugar: sugar or foods/drinks containing sugar	ukb-b-5495	.662	4.437	0.006	3.37E+03	.140	.3625	2.297	0.760	6.942	.321	5.272	0.205	135.802
	Myocardial infarction	ebi-a-GCST011365	Never eat eggs, dairy, wheat, sugar: eggs or foods containing eggs	ukb-b-17455	.348	3.96E+05	0.000	1.62E+16	.789	.976471	0.412	0.001	2.72E+02	.355	0.016	0.000	51.245
			Never eat eggs, dairy, wheat, sugar: dairy products	ukb-b-18909	.646	0.000	0.000	8.36E+13	.269	.448333	0.037	0.000	12.661	.379	0.007	0.000	42.668
			Never eat eggs, dairy, wheat, sugar: I eat all of the above	ukb-b-2393	.031	0.079	0.008	0.748	.000	0	0.185	0.106	0.323	.405	0.494	0.095	2.571
			Never eat eggs, dairy, wheat, sugar: wheat products	ukb-b-3599	.245	0.181	0.016	2.115	.192	.392	0.316	0.056	1.787	.190	0.228	0.034	1.544
			Never eat eggs, dairy, wheat, sugar: sugar or foods/drinks containing sugar	ukb-b-5495	.449	5.416	0.071	414.055	.065	.26	1.950	0.961	3.961	.636	1.565	0.248	9.888
	Myocardial infarction	ebi-a-GCST90038610	Never eat eggs, dairy, wheat, sugar: eggs or foods containing eggs	ukb-b-17455	.151	1.872	0.907	3.865	.732	.976	0.960	0.763	1.210	.508	0.875	0.603	1.269
			Never eat eggs, dairy, wheat, sugar: dairy products	ukb-b-18909	.268	1.756	0.850	3.629	.890	.988889	0.986	0.808	1.203	.532	0.883	0.626	1.247
			Never eat eggs, dairy, wheat, sugar: I eat all of the above	ukb-b-2393	.049	0.927	0.861	0.998	.000	0	0.964	0.946	0.982	.017	0.921	0.862	0.984
			Never eat eggs, dairy, wheat, sugar: wheat products	ukb-b-3599	.325	0.945	0.852	1.048	.196	.392	0.960	0.903	1.021	.153	0.944	0.879	1.013
			Never eat eggs, dairy, wheat, sugar: sugar or foods/drinks containing sugar	ukb-b-5495	.781	1.019	0.894	1.160	.830	.976471	0.998	0.977	1.019	.980	0.999	0.936	1.067
Arrhythmia	Arrhythmia	bbj-a-86	Never eat eggs, dairy, wheat, sugar: eggs or foods containing eggs	ukb-b-17455	.379	0.000	0.000	4.63E+19	.740	.836786	0.243	0.000	1.03E+03	.718	0.124	0.000	3.70E+03
			Never eat eggs, dairy, wheat, sugar: dairy products	ukb-b-18909	.711	1.47E+14	0.000	1.50E+71	.056	.4425	2.88E+04	0.755	1.10E+09	.281	1.54E+04	0.038	6.28E+09
			Never eat eggs, dairy, wheat, sugar: I eat all of the above	ukb-b-2393	.184	0.039	0.000	4.357	.118	.4425	0.516	0.225	1.18E+00	.481	0.464	0.055	3.881
			Never eat eggs, dairy, wheat, sugar: wheat products	ukb-b-3599	.512	1.89E+06	0.000	6.84E+21	.707	.836786	3.577	0.005	2.75E+03	.570	27.063	0.001	7.03E+05
			Never eat eggs, dairy, wheat, sugar: sugar or foods/drinks containing sugar	ukb-b-5495	.078	2.54E+02	0.614	1.05E+05	.112	.4425	2.400	0.814	7.073	.648	1.626	0.205	12.896
	Arrhythmia	ebi-a-GCST90038611	Never eat eggs, dairy, wheat, sugar: eggs or foods containing eggs	ukb-b-17455	.471	1.232	0.729	2.081	.695	.836786	1.027	0.898	1.175	.811	0.971	0.768	1.226
			Never eat eggs, dairy, wheat, sugar: dairy products	ukb-b-18909	.705	0.871	0.468	1.621	.758	.836786	0.975	0.828	1.147	.640	0.928	0.701	1.229
			Never eat eggs, dairy, wheat, sugar: I eat all of the above	ukb-b-2393	.558	1.016	0.964	1.071	.781	.836786	1.002	0.989	1.015	.616	1.013	0.963	1.066
			Never eat eggs, dairy, wheat, sugar: wheat products	ukb-b-3599	.700	1.014	0.950	1.082	.153	.459	1.030	0.989	1.074	.580	1.017	0.962	1.074
			Never eat eggs, dairy, wheat, sugar: sugar or foods/drinks containing sugar	ukb-b-5495	.598	1.031	0.920	1.156	.575	.836786	0.995	0.976	1.013	.884	0.996	0.943	1.052
	Non-cancer illness code, self-reported: heart arrhythmia	ukb-b-3703	Never eat eggs, dairy, wheat, sugar: eggs or foods containing eggs	ukb-b-17455	.28	0.518	0.194	1.382	.118	.4425	0.935	0.859	1.017	.249	0.908	0.788	1.045
			Never eat eggs, dairy, wheat, sugar: dairy products	ukb-b-18909	.713	1.254	0.502	3.133	.585	.836786	1.032	0.921	1.157	.552	1.056	0.908	1.230
			Never eat eggs, dairy, wheat, sugar: I eat all of the above	ukb-b-2393	.527	1.024	0.953	1.100	.393	.836786	1.004	0.994	1.014	.366	1.017	0.981	1.054
			Never eat eggs, dairy, wheat, sugar: wheat products	ukb-b-3599	.854	1.004	0.964	1.046	.205	.5125	1.017	0.991	1.044	.90	1.002	0.969	1.037
			Never eat eggs, dairy, wheat, sugar: sugar or foods/drinks containing sugar	ukb-b-5495	.533	0.971	0.887	1.064	.896	.896	1.001	0.990	1.011	.451	0.987	0.954	1.021
Myocarditis	Myocarditis	ebi-a-GCST90018882	Never eat eggs, dairy, wheat, sugar: eggs or foods containing eggs	ukb-b-17455	.532	0.000	0.000	3.79E+26	.359	.8295	0.000	0.000	2.01E+06	.588	0.000	0.000	5.34E+14
			Never eat eggs, dairy, wheat, sugar: dairy products	ukb-b-18909	.132	0.000	0.000	1.39E−12	.431	.8295	0.000	0.000	1.89E+11	.995	1.224	0.000	1.39E+27
			Never eat eggs, dairy, wheat, sugar: I eat all of the above	ukb-b-2393	.582	58.964	0.000	1.10E+08	.539	.8295	2.695	0.114	6.40E+01	.566	0.061	0.000	8.03E+02
			Never eat eggs, dairy, wheat, sugar: wheat products	ukb-b-3599	.358	2778.439	0.000	1.70E+10	.654	.891818	13.348	0.000	1.13E+06	.493	112.693	0.000	4.05E+07
			Never eat eggs, dairy, wheat, sugar: sugar or foods/drinks containing sugar	ukb-b-5495	.238	2.54E+05	0.000	1.86E+14	.899	.976	1.250	0.040	39.198	.357	198.661	0.003	1.39E+07
	Myocarditis	finn-b-I9_MYOCARD	Never eat eggs, dairy, wheat, sugar: eggs or foods containing eggs	ukb-b-17455	.651	0.000	0.000	5.54E+26	.100	.8295	0.000	0.000	62.321	.245	0.000	0.000	2.60E+06
			Never eat eggs, dairy, wheat, sugar: dairy products	ukb-b-18909	.202	0.000	0.000	4.66E+01	.450	.8295	0.000	0.000	1.30E+09	.732	8.09E+04	0.000	3.14E+30
			Never eat eggs, dairy, wheat, sugar: I eat all of the above	ukb-b-2393	.821	4.978	0.000	5.29E+06	.976	.976	1.049	0.048	2.29E+01	.965	1.223	0.000	8.96E+03
			Never eat eggs, dairy, wheat, sugar: wheat products	ukb-b-3599	.367	1.98E+05	0.000	3.35E+15	.553	.8295	112.192	0.000	6.55E+08	.213	1.70E+04	0.026	1.11E+10
			Never eat eggs, dairy, wheat, sugar: sugar or foods/drinks containing sugar	ukb-b-5495	.509	615.850	0.000	1.04E+11	.197	.8295	0.123	0.005	2.97E+00	.642	0.083	0.000	2.87E+03
	Myocarditis	ebi-a-GCST90018662	Never eat eggs, dairy, wheat, sugar: eggs or foods containing eggs	ukb-b-17455	.140	1.50E+50	0.000	########	.480	.8295	1.23E+06	0.000	1.02E+23	.536	9.18E+08	0.000	7.02E+36
			Never eat eggs, dairy, wheat, sugar: dairy products	ukb-b-18909	.465	0.000	0.000	3.05E+87	.923	.976	0.051	0.000	9.18E+24	.745	0.000	0.000	9.35E+36
			Never eat eggs, dairy, wheat, sugar: I eat all of the above	ukb-b-2393	.124	1.21E+10	0.002	6.67E+22	.229	.8295	83.417	0.062	1.13E+05	.728	34.376	0.000	1.52E+10
			Never eat eggs, dairy, wheat, sugar: wheat products	ukb-b-3599	.546	2.12E+03	0.000	9.01E+13	.749	.93625	0.043	0.000	9.96E+06	.953	1.904	0.000	3.66E+09
			Never eat eggs, dairy, wheat, sugar: sugar or foods/drinks containing sugar	ukb-b-5495	.481	1.22E+05	0.000	1.56E+19	.492	.8295	0.064	0.000	1.64E+02	.713	55.026	0.000	9.82E+10
Cardiomyopathy	Cardiomyopathy	finn-b-I9_CARDMYO	Never eat eggs, dairy, wheat, sugar: eggs or foods containing eggs	ukb-b-17455	.991	1.324	0.000	2.47E+19	.116	.368571	6.44E+04	0.065	6.39E+10	.464	8.49E+04	0.000	1.28E+17
			Never eat eggs, dairy, wheat, sugar: dairy products	ukb-b-18909	.308	0.000	0.000	2.18E+06	.338	.563333	4.28E+03	0.000	1.17E+11	.840	0.050	0.000	2.19E+10
			Never eat eggs, dairy, wheat, sugar: I eat all of the above	ukb-b-2393	.960	1.224	0.000	3.32E+03	.107	.368571	0.237	0.041	1.364	.707	0.313	0.001	1.32E+02
			Never eat eggs, dairy, wheat, sugar: wheat products	ukb-b-3599	.991	0.942	0.000	1.80E+04	.160	.368571	0.008	0.000	6.682	.702	0.203	0.000	4.48E+02
			Never eat eggs, dairy, wheat, sugar: sugar or foods/drinks containing sugar	ukb-b-5495	.533	0.038	0.000	1.05E+03	.235	.440625	2.840	0.508	1.59E+01	.860	0.654	0.006	7.10E+01
	Cardiomyopathy (excluding other)	finn-b-FG_CARDMYO	Never eat eggs, dairy, wheat, sugar: eggs or foods containing eggs	ukb-b-17455	.942	0.139	0.000	8.03E+20	.156	.368571	8.17E+04	0.014	4.91E+11	.261	2.88E+08	0.000	3.63E+21
			Never eat eggs, dairy, wheat, sugar: dairy products	ukb-b-18909	.773	0.000	0.000	2.07E+19	.172	.368571	1.83E+05	0.005	6.57E+12	.702	256.192	0.000	4.04E+13
			Never eat eggs, dairy, wheat, sugar: I eat all of the above	ukb-b-2393	.945	0.769	0.000	1.22E+03	.535	.617143	0.533	0.073	3.89E+00	.789	0.437	0.001	1.86E+02
			Never eat eggs, dairy, wheat, sugar: wheat products	ukb-b-3599	.760	0.140	0.000	1.87E+04	.684	.684	0.207	0.000	4.09E+02	.847	0.414	0.000	2.01E+03
			Never eat eggs, dairy, wheat, sugar: sugar or foods/drinks containing sugar	ukb-b-5495	.132	0.010	0.000	3.673	.532	.617143	1.766	0.296	1.05E+01	.784	0.473	0.002	9.71E+01
	Cardiomyopathy, other and unspecified	finn-b-I9_CARDMYOOTH	Never eat eggs, dairy, wheat, sugar: eggs or foods containing eggs	ukb-b-17455	.573	1.34E+09	0.000	1.97E+38	.411	.6165	6.44E+03	0.000	7.87E+12	.801	0.004	0.000	1.16E+15
			Never eat eggs, dairy, wheat, sugar: dairy products	ukb-b-18909	.373	0.000	0.000	1.10E+13	.070	.368571	1.16E+11	0.126	1.06E+23	.657	1.08E+04	0.000	1.34E+20
			Never eat eggs, dairy, wheat, sugar: I eat all of the above	ukb-b-2393	.128	7.26E+02	0.169	3.12E+06	.470	.617143	0.433	0.045	4.200	.526	9.641	0.009	1.02E+04
			Never eat eggs, dairy, wheat, sugar: wheat products	ukb-b-3599	.649	0.024	0.000	########	.094	.368571	0.000	0.000	4.309	.420	0.006	0.000	5.17E+02
			Never eat eggs, dairy, wheat, sugar: sugar or foods/drinks containing sugar	ukb-b-5495	.095	0.001	0.000	2.891	.576	.617143	0.505	0.046	5.532	.885	0.564	0.000	1.31E+03
Valvular heart disease	Heart valve problem or heart murmur	ebi-a-GCST90038612	Never eat eggs, dairy, wheat, sugar: eggs or foods containing eggs	ukb-b-17455	.395	0.861	0.628	1.180	.969	.997	0.998	0.918	1.086	.669	0.965	0.827	1.127
			Never eat eggs, dairy, wheat, sugar: dairy products	ukb-b-18909	.570	0.862	0.560	1.327	.250	.857111	0.933	0.829	1.050	.428	0.915	0.757	1.106
			Never eat eggs, dairy, wheat, sugar: I eat all of the above	ukb-b-2393	.137	1.025	0.992	1.059	.287	.857111	1.005	0.996	1.015	.431	1.01	0.981	1.047
			Never eat eggs, dairy, wheat, sugar: wheat products	ukb-b-3599	.449	1.020	0.973	1.069	.751	.997	1.005	0.975	1.035	.535	1.011	0.978	1.045
			Never eat eggs, dairy, wheat, sugar: sugar or foods/drinks containing sugar	ukb-b-5495	.878	1.003	0.964	1.044	.436	.857111	0.996	0.985	1.007	.930	0.998	0.962	1.036
	Non-cancer illness code, self-reported: heart valve problem/heart murmur	ukb-b-18578	Never eat eggs, dairy, wheat, sugar: eggs or foods containing eggs	ukb-b-17455	.669	0.768	0.257	2.292	.910	.997	1.005	0.915	1.105	.622	1.043	0.893	1.218
			Never eat eggs, dairy, wheat, sugar: dairy products	ukb-b-18909	.851	0.883	0.318	2.449	.510	.857111	0.958	0.844	1.088	.510	0.935	0.791	1.104
			Never eat eggs, dairy, wheat, sugar: I eat all of the above	ukb-b-2393	.141	1.053	0.984	1.127	.993	.997	1.000	0.990	1.010	.898	1.003	0.965	1.042
			Never eat eggs, dairy, wheat, sugar: wheat products	ukb-b-3599	.355	1.024	0.979	1.071	.157	.857111	1.021	0.992	1.051	.252	1.023	0.988	1.058
			Never eat eggs, dairy, wheat, sugar: sugar or foods/drinks containing sugar	ukb-b-5495	.265	0.952	0.875	1.037	.551	.857111	0.997	0.985	1.008	.769	0.995	0.965	1.026
	Valvular heart disease including rheumatic fever	finn-b-I9_VHD	Never eat eggs, dairy, wheat, sugar: eggs or foods containing eggs	ukb-b-17455	.361	2.63E+03	0.001	8.40E+09	.526	.857111	0.200	0.001	28.800	.484	0.027	0.000	3.14E+02
			Never eat eggs, dairy, wheat, sugar: dairy products	ukb-b-18909	.517	5.88E+02	0.000	5.40E+09	.435	.857111	0.125	0.001	23.147	.911	1.715	0.000	1.08E+04
			Never eat eggs, dairy, wheat, sugar: I eat all of the above	ukb-b-2393	.437	2.548	0.244	2.66E+01	.004	.056	0.460	0.272	0.777	.253	0.338	0.054	2.132
			Never eat eggs, dairy, wheat, sugar: wheat products	ukb-b-3599	.416	5.396	0.140	2.08E+02	.997	.997	0.995	0.073	13.573	.524	2.452	0.187	32.075
			Never eat eggs, dairy, wheat, sugar: sugar or foods/drinks containing sugar	ukb-b-5495	.029	0.017	0.000	0.591	.482	.663667	1.252	0.669	2.344	.775	1.351	0.173	10.529
Cardiac death	Death due to cardiac causes	finn-b-I9_K_CARDIAC	Never eat eggs, dairy, wheat, sugar: eggs or foods containing eggs	ukb-b-17455	.628	3815.820	0.000	9.40E+16	.134	.306167	1.47E+3	0.105	2.07E+07	.505	484.324	0.000	1.05E+10
			Never eat eggs, dairy, wheat, sugar: dairy products	ukb-b-18909	.901	10.393	0.000	1.49E+15	.673	.673	9.77E+00	0.000	3.88E+05	.943	1.876	0.000	1.40E+07
			Never eat eggs, dairy, wheat, sugar: I eat all of the above	ukb-b-2393	.594	0.334	0.006	1.84E+01	.019	.069667	0.277	0.095	8.10E−01	.467	0.245	0.006	1.06E+01
			Never eat eggs, dairy, wheat, sugar: wheat products	ukb-b-3599	.073	4.41E+03	4.907	3.97E+06	.167	.306167	41.139	0.212	8.00E+03	.140	142.287	0.555	3.65E+04
			Never eat eggs, dairy, wheat, sugar: sugar or foods/drinks containing sugar	ukb-b-5495	.695	0.431	0.007	2.82E+01	.154	.306167	2.496	0.710	8.784	.551	2.939	0.087	9.97E+01
	Death due to cardiac causes	ukb-d-I9_K_CARDIAC	Never eat eggs, dairy, wheat, sugar: eggs or foods containing eggs	ukb-b-17455	.321	1.164	0.888	1.526	.003	.0165	1.116	1.038	1.201	.183	1.098	0.972	1.240
			Never eat eggs, dairy, wheat, sugar: dairy products	ukb-b-18909	.783	0.927	0.576	1.491	.615	.673	0.973	0.873	1.084	.661	0.955	0.794	1.150
			Never eat eggs, dairy, wheat, sugar: I eat all of the above	ukb-b-2393	.012	0.965	0.939	0.992	.003	.0165	0.988	0.980	0.996	.859	0.998	0.973	1.023
			Never eat eggs, dairy, wheat, sugar: wheat products	ukb-b-3599	.836	1.005	0.958	1.055	.543	.663667	1.010	0.979	1.042	.486	1.013	0.979	1.048
			Never eat eggs, dairy, wheat, sugar: sugar or foods/drinks containing sugar	ukb-b-5495	.934	0.999	0.964	1.034	.489	.664	1.003	0.994	1.013	.839	0.997	0.966	1.028

FDR = false discovery rate, MR = Mendelian randomization.

### 3.3. Meta-analysis

Based on the results of TSMR performed with dietary habits as exposure and multiple CVDs as the outcome obtained in Step 1, a meta-analysis was conducted according to various CVD classifications. The meta-analysis results are presented in Table 2 and Figure S11, Supplemental Digital Content, https://links.lww.com/MD/P886. Our study found a positive association between “Never eat eggs” and cardiomyopathy (OR = 44,563; 95% CI = 4.194–66,844.4; *P* = .024) as well as cardiac death (OR = 1.117; 95% CI = 1.038–1.201; *P* = .003). Similarly, “Never eat dairy products” was positively associated with essential hypertension (OR = 1.022; 95% CI = 1.004–1.04; *P* = .003). “Never eat sugar or foods/drinks containing sugar” was positively associated with HF (OR = 3.099; 95% CI = 1.776–5.408; meta-*P* < .001) and CHD (OR = 1.705; 95% CI = 1.175–2.474; meta-*P* = .005). In contrast, a balanced diet (I eat all of the above) was negatively associated with HF (OR = 0.194; 95% CI = 0.128–0.294; meta-*P* < .001), CHD (OR = 0.2; 95% CI = 0.133–0.301; meta-*P* < .001), and MI (OR = 0.96; 95% CI = 0.942–0.978; meta-*P* < .001).

**Table 2:** Meta-analysis of cardiovascular TSMR results from different databases.

Disease	Exposure-name	Exposure-ID	Outcome-name	Outcome-ID	Meta-analysis								
					Fixed effect model				Random effect model				Sensitivity analysis
					OR	or_lci95	or_uci95	*P*	OR	or_lci95	or_uci95	*P*	*P*
Heart failure	Never eat eggs, dairy, wheat, sugar: eggs or foods containing eggs	ukb-b-17455	Chronic heart failure	ebi-a-GCST90018806	5.547	0.067	8.32	.447	5.547	0.067	8.32	.447	<.001
			All-cause heart Failure	finn-b-I9_HEARTFAIL_ALLCAUSE									
			Heart failure, strict	finn-b-I9_HEARTFAIL									
	Never eat eggs, dairy, wheat, sugar: dairy products	ukb-b-18909	Chronic heart failure	ebi-a-GCST90018806	82.006	0.917	123.008	.055	82.006	0.917	123.008	.055	.6946
			All-cause heart Failure	finn-b-I9_HEARTFAIL_ALLCAUSE									
			Heart failure, strict	finn-b-I9_HEARTFAIL									
	Never eat eggs, dairy, wheat, sugar: I eat all of the above	ukb-b-2393	Chronic heart failure	ebi-a-GCST90018806	0.194	0.128	0.294	0	0.194	0.128	0.294	0	.6646
			All-cause heart failure	finn-b-I9_HEARTFAIL_ALLCAUSE									
			Heart failure, strict	finn-b-I9_HEARTFAIL									
	Never eat eggs, dairy, wheat, sugar: wheat products	ukb-b-3599	Chronic heart failure	ebi-a-GCST90018806	0.624	0.151	2.587	.516	0.838	0.122	5.78	.858	.2471
			All-cause heart failure	finn-b-I9_HEARTFAIL_ALLCAUSE									
			Heart failure, strict	finn-b-I9_HEARTFAIL									
	Never eat eggs, dairy, wheat, sugar: sugar or foods/drinks containing sugar	ukb-b-5495	Chronic heart failure	ebi-a-GCST90018806	3.099	1.776	5.408	0	3.099	1.776	5.408	0	.8359
			All-cause heart failure	finn-b-I9_HEARTFAIL_ALLCAUSE									
			Heart failure, strict	finn-b-I9_HEARTFAIL									
Essential hypertension	Never eat eggs, dairy, wheat, sugar: eggs or foods containing eggs	ukb-b-17455	Diagnoses – secondary ICD10: I10 Essential (primary) hypertension	ukb-b-12493	0.994	0.955	1.035	.784	0.994	0.955	1.035	.784	.5507
			Non-cancer illness code, self-reported: essential hypertension	ukb-b-7582									
			Essential hypertension	ebi-a-GCST90038608									
	Never eat eggs, dairy, wheat, sugar: dairy products	ukb-b-18909	Diagnoses – secondary ICD10: I10 Essential (primary) hypertension	ukb-b-12493	1.002	0.944	1.063	.95	1.002	0.944	1.063	.95	.4064
			Non-cancer illness code, self-reported: essential hypertension	ukb-b-7582									
			Essential hypertension	ebi-a-GCST90038608									
	Never eat eggs, dairy, wheat, sugar: I eat all of the above	ukb-b-2393	Diagnoses – secondary ICD10: I10 Essential (primary) hypertension	ukb-b-12493	0.996	0.991	1.001	.091	0.945	0.848	1.053	.307	<.001
			Non-cancer illness code, self-reported: essential hypertension	ukb-b-7582									
			Essential hypertension	ebi-a-GCST90038608									
	Never eat eggs, dairy, wheat, sugar: wheat products	ukb-b-3599	Diagnoses – secondary ICD10: I10 Essential (primary) hypertension	ukb-b-12493	1.022	1.004	1.04	.017	1.025	1	1.05	.053	.2376
			Non-cancer illness code, self-reported: essential hypertension	ukb-b-7582									
			Essential hypertension	ebi-a-GCST90038608									
	Never eat eggs, dairy, wheat, sugar: sugar or foods/drinks containing sugar	ukb-b-5495	Diagnoses – secondary ICD10: I10 Essential (primary) hypertension	ukb-b-12493	1.011	1.004	1.018	.002	1.048	0.968	1.135	.243	<.0001
			Non-cancer illness code, self-reported: essential hypertension	ukb-b-7582									
			Essential hypertension	ebi-a-GCST90038608									
Coronary heart disease	Never eat eggs, dairy, wheat, sugar: eggs or foods containing eggs	ukb-b-17455	Coronary artery disease	ebi-a-GCST005195	0.066	0.003	1.698	.101	0.066	0.003	1.698	.101	.8867
			Coronary artery disease (Firth correction)	ebi-a-GCST90013864									
			Coronary heart disease	ieu-a-7									
	Never eat eggs, dairy, wheat, sugar: dairy products	ukb-b-18909	Coronary artery disease	ebi-a-GCST005195	0.198	0.005	8.38	.397	0.198	0.005	8.38	.397	.8124
			Coronary artery disease (Firth correction)	ebi-a-GCST90013864									
			Coronary heart disease	ieu-a-7									
	Never eat eggs, dairy, wheat, sugar: I eat all of the above	ukb-b-2393	Coronary artery disease	ebi-a-GCST005195	0.2	0.133	0.301	0	0.2	0.133	0.301	0	.5478
			Coronary artery disease (Firth correction)	ebi-a-GCST90013864									
			Coronary heart disease	ieu-a-7									
	Never eat eggs, dairy, wheat, sugar: wheat products	ukb-b-3599	Coronary artery disease	ebi-a-GCST005195	0.575	0.227	1.458	.243	0.575	0.227	1.458	.243	.6426
			Coronary artery disease (Firth correction)	ebi-a-GCST90013864									
			Coronary heart disease	ieu-a-7									
	Never eat eggs, dairy, wheat, sugar: sugar or foods/drinks containing sugar	ukb-b-5495	Coronary artery disease	ebi-a-GCST005195	1.705	1.175	2.474	.005	1.705	1.175	2.474	.005	.7915
			Coronary artery disease (Firth correction)	ebi-a-GCST90013864									
			Coronary heart disease	ieu-a-7									
Myocardial infarction	Never eat eggs, dairy, wheat, sugar: eggs or foods containing eggs	ukb-b-17455	Myocardial infarction	ebi-a-GCST90018877	0.96	0.726	1.208	.727	0.96	0.726	1.208	.727	.995
			Myocardial infarction	finn-b-I9_MI									
			Myocardial infarction	ebi-a-GCST011365									
			Myocardial infarction	ebi-a-GCST90038610									
	Never eat eggs, dairy, wheat, sugar: dairy products	ukb-b-18909	Myocardial infarction	ebi-a-GCST90018877	0.977	0.801	1.192	.819	0.325	0.03	3.571	.358	.3837
			Myocardial infarction	finn-b-I9_MI									
			Myocardial infarction	ebi-a-GCST011365									
			Myocardial infarction	ebi-a-GCST90038610									
	Never eat eggs, dairy, wheat, sugar: I eat all of the above	ukb-b-2393	Myocardial infarction	ebi-a-GCST90018877	0.96	0.942	0.978	0	0.308	0.128	0.743	.009	<.001
			Myocardial infarction	finn-b-I9_MI									
			Myocardial infarction	ebi-a-GCST011365									
			Myocardial infarction	ebi-a-GCST90038610									
	Never eat eggs, dairy, wheat, sugar: wheat products	ukb-b-3599	Myocardial infarction	ebi-a-GCST90018877	0.958	0.902	1.019	.174	0.687	0.258	1.827	.451	.0961
			Myocardial infarction	finn-b-I9_MI									
			Myocardial infarction	ebi-a-GCST011365									
			Myocardial infarction	ebi-a-GCST90038610									
	Never eat eggs, dairy, wheat, sugar: sugar or foods/drinks containing sugar	ukb-b-5495	Myocardial infarction	ebi-a-GCST90018877	0.999	0.978	1.02	.912	1.366	0.877	2.127	.168	.0937
			Myocardial infarction	finn-b-I9_MI									
			Myocardial infarction	ebi-a-GCST011365									
			Myocardial infarction	ebi-a-GCST90038610									
Arrhythmia	Never eat eggs, dairy, wheat, sugar: eggs or foods containing eggs	ukb-b-17455	Arrhythmia	bbj-a-86	0.96	0.894	1.031	.264	0.965	0.884	1.053	.426	.4814
			Arrhythmia	ebi-a-GCST90038611									
			Non-cancer illness code, self-reported: heart arrhythmia	ukb-b-3703									
	Never eat eggs, dairy, wheat, sugar: dairy products	ukb-b-18909	Arrhythmia	bbj-a-86	1.014	0.923	1.113	.774	1.014	0.923	1.113	.774	.1388
			Arrhythmia	ebi-a-GCST90038611									
			Non-cancer illness code, self-reported: heart arrhythmia	ukb-b-3703									
	Never eat eggs, dairy, wheat, sugar: I eat all of the above	ukb-b-2393	Arrhythmia	bbj-a-86	1.003	0.995	1.011	.404	1.003	0.995	1.011	.405	.2796
			Arrhythmia	ebi-a-GCST90038611									
			Non-cancer illness code, self-reported: heart arrhythmia	ukb-b-3703									
	Never eat eggs, dairy, wheat, sugar: wheat products	ukb-b-3599	Arrhythmia	bbj-a-86	1.021	0.999	1.043	.066	1.021	0.999	1.043	.066	.8122
			Arrhythmia	ebi-a-GCST90038611									
			Non-cancer illness code, self-reported: heart arrhythmia	ukb-b-3703									
	Never eat eggs, dairy, wheat, sugar: sugar or foods/drinks containing sugar	ukb-b-5495	Arrhythmia	bbj-a-86	0.999	0.99	1.009	.879	0.999	0.99	1.009	.878	.2431
			Arrhythmia	ebi-a-GCST90038611									
			Non-cancer illness code, self-reported: heart arrhythmia	ukb-b-3703									
Myocarditis	Never eat eggs, dairy, wheat, sugar: eggs or foods containing eggs	ukb-b-17455	Myocarditis	ebi-a-GCST90018882	0	0	0	.179	0	0	0	.179	.3271
			Myocarditis	finn-b-I9_MYOCARD									
			Myocarditis	ebi-a-GCST90018662									
	Never eat eggs, dairy, wheat, sugar: dairy products	ukb-b-18909	Myocarditis	ebi-a-GCST90018882	0	0	0	.305	0	0	0	.305	.93
			Myocarditis	finn-b-I9_MYOCARD									
			Myocarditis	ebi-a-GCST90018662									
	Never eat eggs, dairy, wheat, sugar: I eat all of the above	ukb-b-2393	Myocarditis	ebi-a-GCST90018882	2.323	0.281	19.194	.434	2.323	0.281	19.194	.434	.5457
			Myocarditis	finn-b-I9_MYOCARD									
			Myocarditis	ebi-a-GCST90018662									
	Never eat eggs, dairy, wheat, sugar: wheat products	ukb-b-3599	Myocarditis	ebi-a-GCST90018882	8.431	0.002	12.646	.614	8.431	0.002	12.646	.614	.8183
			Myocarditis	finn-b-I9_MYOCARD									
			Myocarditis	ebi-a-GCST90018662									
	Never eat eggs, dairy, wheat, sugar: sugar or foods/drinks containing sugar	ukb-b-5495	Myocarditis	ebi-a-GCST90018882	0.311	0.033	2.925	.307	0.311	0.033	2.925	.307	.5739
			Myocarditis	finn-b-I9_MYOCARD									
			Myocarditis	ebi-a-GCST90018662									
Cardiomyopathy	Never eat eggs, dairy, wheat, sugar: eggs or foods containing eggs	ukb-b-17455	Cardiomyopathy	finn-b-I9_CARDMYO	44,562.91	4.194	66,844.36	.024	44,562.91	4.194	66,844.36	.024	.9795
			Cardiomyopathy (excluding other)	finn-b-FG_CARDMYO									
			Cardiomyopathy, other and unspecified	finn-b-I9_CARDMYOOTH									
	Never eat eggs, dairy, wheat, sugar: dairy products	ukb-b-18909	Cardiomyopathy	finn-b-I9_CARDMYO	3,32,559.8	4.745	4,98,839.7	.026	3,32,559.8	4.745	4,98,839.7	.026	.5836
			Cardiomyopathy (excluding other)	finn-b-FG_CARDMYO									
			Cardiomyopathy, other and unspecified	finn-b-I9_CARDMYOOTH									
	Never eat eggs, dairy, wheat, sugar: I eat all of the above	ukb-b-2393	Cardiomyopathy	finn-b-I9_CARDMYO	0.359	0.115	1.121	.078	0.359	0.115	1.121	.078	.8206
			Cardiomyopathy (excluding other)	finn-b-FG_CARDMYO									
			Cardiomyopathy, other and unspecified	finn-b-I9_CARDMYOOTH									
	Never eat eggs, dairy, wheat, sugar: wheat products	ukb-b-3599	Cardiomyopathy	finn-b-I9_CARDMYO	0.012	0	1.072	.054	0.012	0	1.072	.054	.5413
			Cardiomyopathy (excluding other)	finn-b-FG_CARDMYO									
			Cardiomyopathy, other and unspecified	finn-b-I9_CARDMYOOTH									
	Never eat eggs, dairy, wheat, sugar: sugar or foods/drinks containing sugar	ukb-b-5495	Cardiomyopathy	finn-b-I9_CARDMYO	1.646	0.548	4.946	.375	1.646	0.548	4.946	.375	.5147
			Cardiomyopathy (excluding other)	finn-b-FG_CARDMYO									
			Cardiomyopathy, other and unspecified	finn-b-I9_CARDMYOOTH									
Valvular heart disease	Never eat eggs, dairy, wheat, sugar: eggs or foods containing eggs	ukb-b-17455	Heart valve problem or heart murmur	ebi-a-GCST90038612	1.001	0.94	1.066	.969	1.001	0.94	1.066	.969	.8126
			Non-cancer illness code, self-reported: heart valve problem/heart murmur	ukb-b-18578									
			Valvular heart disease including rheumatic fever	finn-b-I9_VHD									
	Never eat eggs, dairy, wheat, sugar: dairy products	ukb-b-18909	Heart valve problem or heart murmur	ebi-a-GCST90038612	0.944	0.866	1.029	.192	0.944	0.866	1.029	.192	.7162
			Non-cancer illness code, self-reported: heart valve problem/heart murmur	ukb-b-18578									
			Valvular heart disease including rheumatic fever	finn-b-I9_VHD									
	Never eat eggs, dairy, wheat, sugar: I eat all of the above	ukb-b-2393	Heart valve problem or heart murmur	ebi-a-GCST90038612	1.003	0.996	1.01	.457	1.003	0.996	1.01	.457	.011
			Non-cancer illness code, self-reported: heart valve problem/heart murmur	ukb-b-18578									
			Valvular heart disease including rheumatic fever	finn-b-I9_VHD									
	Never eat eggs, dairy, wheat, sugar: wheat products	ukb-b-3599	Heart valve problem or heart murmur	ebi-a-GCST90038612	1.013	0.992	1.034	.215	1.013	0.992	1.034	.215	.7536
			Non-cancer illness code, self-reported: heart valve problem/heart murmur	ukb-b-18578									
			Valvular heart disease including rheumatic fever	finn-b-I9_VHD									
	Never eat eggs, dairy, wheat, sugar: sugar or foods/drinks containing sugar	ukb-b-5495	Heart valve problem or heart murmur	ebi-a-GCST90038612	0.996	0.988	1.004	.334	0.996	0.988	1.004	.334	.7705
			Non-cancer illness code, self-reported: heart valve problem/heart murmur	ukb-b-18578									
			Valvular heart disease including rheumatic fever	finn-b-I9_VHD									
Cardiac death	Never eat eggs, dairy, wheat, sugar: eggs or foods containing eggs	ukb-b-17455	Death due to cardiac causes	finn-b-I9_K_CARDIAC	1.117	1.038	1.201	.003	7.76	0.015	11.64	.52	.1405
			Death due to cardiac causes	ukb-d-I9_K_CARDIAC									
	Never eat eggs, dairy, wheat, sugar: dairy products	ukb-b-18909	Death due to cardiac causes	finn-b-I9_K_CARDIAC	0.973	0.873	1.084	.618	0.973	0.873	1.084	.618	.6694
			Death due to cardiac causes	ukb-d-I9_K_CARDIAC									
	Never eat eggs, dairy, wheat, sugar: I eat all of the above	ukb-b-2393	Death due to cardiac causes	finn-b-I9_K_CARDIAC	0.988	0.98	0.996	.003	0.589	0.173	2.001	.396	.0201
			Death due to cardiac causes	ukb-d-I9_K_CARDIAC									
	Never eat eggs, dairy, wheat, sugar: wheat products	ukb-b-3599	Death due to cardiac causes	finn-b-I9_K_CARDIAC	1.01	0.979	1.042	.538	2.431	0.111	53.407	.573	.168
			Death due to cardiac causes	ukb-d-I9_K_CARDIAC									
	Never eat eggs, dairy, wheat, sugar: sugar or foods/drinks containing sugar	ukb-b-5495	Death due to cardiac causes	finn-b-I9_K_CARDIAC	1.003	0.994	1.013	.482	1.263	0.581	2.743	.556	.1556
			Death due to cardiac causes	ukb-d-I9_K_CARDIAC									

OR = odds ratio, TSMR = two-sample Mendelian randomization.

### 3.4. Co-localization analysis and bioinformatics analysis

**Figure 2.**
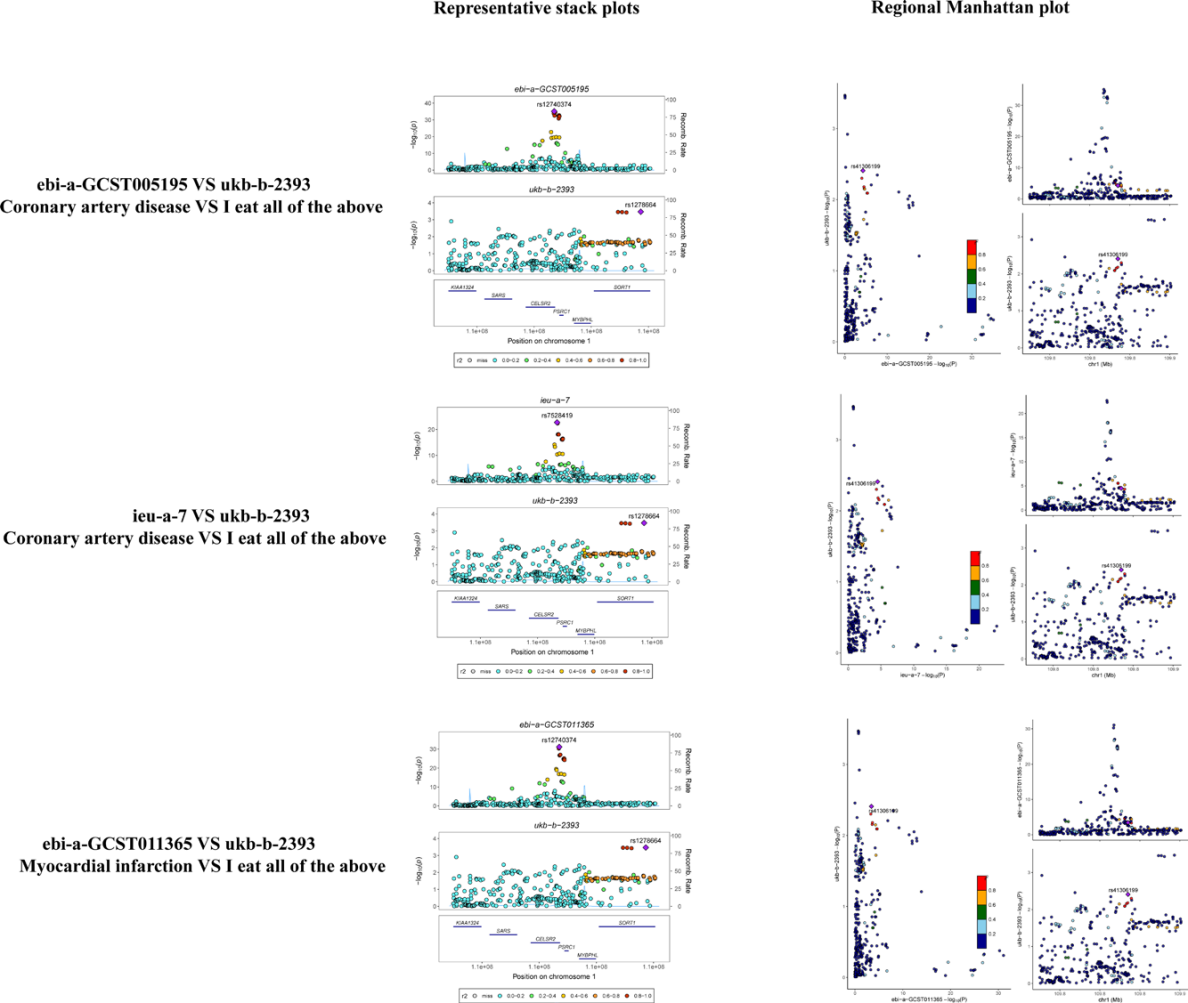
Co-location analysis results.

**Figure 3.**
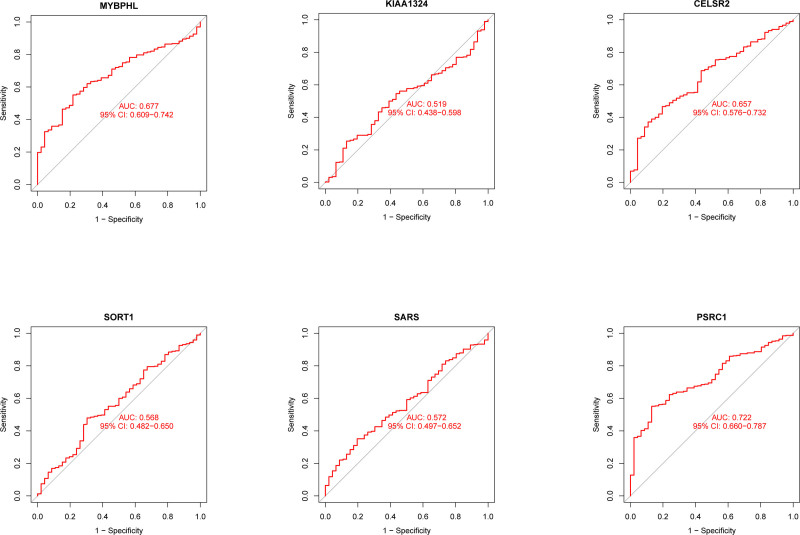
ROC curves based on the risk model. AUC = area under the curve, CI = confidence interval.

Based on the Step 1 and Step 2 results, we subjected all positive results to co-localization analysis. Our results (see Table 3) indicate that SNP rs12740374 (PP.H4 = 0.855) drives the causal variance of CHD and balanced diet. In addition, SNP rs7528419 (PP.H4 = 0.716) drives the causal variance of MI and a balanced diet. It is noteworthy that 2 SNPs, rs7528419 and rs12740374 (Fig. 2), drive the common motifs of 6 genes: KIAA1324, SARS, CELSR2, PSRC1, MYBPHL, and SORT1. Based on GeneChip GSE59867, our study investigated the association of 6 genes with CHD/MI. ROC curves based on the risk model (Fig. 3), demonstrated that PSRC1 (AUC: 0.722, 95% CI 0.66–0.787), CELSR2 (AUC: 0.657, 95% CI 0.576–0.732), and MYBPHL (AUC: 0.677, 95% CI 0.609–0.742) have high diagnostic value for CHD and MI.

**Table 3:** Co-localization analysis results.

Co-localization-ID	SNP	V.df1	z.df1	r.df1	lABF.df1	V.df2	z.df2	r.df2	lABF.df2	internal.sum.lABF	SNP.PP.H4
ebi-a-GCST011365;ukb-b-2393	rs12740374	8.12E−05	−11.74203928	0.997908885	65.70855752	1.10E−06	−0.693020819	0.999722037	−3.853938267	61.85461925	0.854635972
ieu-a-7;ukb-b-2393	rs7528419	0.000131836	−9.974743076	0.999325679	46.06330165	1.10E−06	−0.799804585	0.999722868	−3.775752874	42.28754878	0.716385554

SNP = ingle nucleotide polymorphism.

## 4. Discussion

The Healthy Eating Index (HEI)^[5]^ was developed in the United States based on the health benefits of a balanced diet. It provides recommendations and guidelines for Americans and individuals worldwide regarding the consumption of various types of foods, including fruits, vegetables, grains, and sugars. Not everyone maintains a balanced diet. In light of increasing attention to diet and health, an increasing number of individuals hold the belief that a diet high in carbohydrates (such as sugar and wheat) negatively impacts cardiovascular health.^[7]^ Furthermore, studies indicate a positive correlation between carbohydrate intake and the risk of CVD,^[18]^ bolstering the belief that reducing carbohydrate intake could help lower the incidence of such disease.^[19]^ Numerous studies have demonstrated that a depletion of carbohydrates, resulting in increased metabolism of ketone bodies, represents a crucial characteristic of HF.^[20]^ This process could be attributed to the function of G protein-coupled receptors and histone deacetylases, which regulate metabolism and gene expression. However, it has been demonstrated that supplementing additional ketone bodies during HF can decrease its severity.^[21]^ This may be due to the fact that ketone body oxidation stimulates endothelial cell proliferation and neovascularization,^[22]^ suggesting a novel therapeutic approach for HF. Similarly, some scholars suggest a low-fat, high-carbohydrate diet for patients with HF or hypertension. This may be due to the fact that a high-carbohydrate diet can activate the PPAR system, leading to an increase in cardiac gene expression and cell growth.^[8]^ Additionally, studies on egg consumption have found significant correlations with both CVD events and all-cause mortality.^[10]^ There is also evidence to suggest that older adults who consume 5–6 eggs/wk have a 30% higher risk of CHD (95% CI: 3–56%).^[23]^ It is widely recognized that whole milk is considered unhealthy due to its high-fat content. Furthermore, decreasing the consumption of unhealthy foods reduces the chances of CHD death or nonfatal MI, as well as the risk of diabetes.^[24]^ Related research guidelines recommend that individuals with CVD should decrease their consumption of dairy products like cream and butter, limit their intake of sugary foods and beverages, and lower intake of cholesterol-rich foods such as eggs.^[25,26]^

Our study supports the strong correlation between not eating eggs or foods containing eggs and the incidence of cardiovascular health. We found a significant positive association between “Never eat eggs or foods containing eggs” and a higher incidence of cardiomyopathy (OR = 44,563; 95% CI = 4.194–66,844.4; meta-*P* = .024) as well as cardiac death (OR = 1.117; 95% CI = 1.038–1.201; meta-*P* = .003). In addition, our findings suggest the conclusion that not eating dairy products may be detrimental to cardiovascular health. In fact, our results showing a positive association between consuming “Never eat dairy products” and essential hypertension (OR = 1.022; 95% CI = 1.004–1.04; meta-*P* = .003). Furthermore, our findings do not wholly corroborate the hypothesis that the consumption of carbohydrates derived from wheat or sugar is a significant risk factor for the development of CVD. The present study on carbohydrates revealed that “Never eat sugar” or “Never eat sugar” were positively associated with HF (OR = 3.099; 95% CI = 1.776–5.408; meta-*P* < .001) and CHD (OR = 1.705; 95% CI = 1.175–2.474; meta-*P* = .005). In contrast, the study on wheat products found no significant association between wheat product consumption and the discussed CVD risks. In contrast to these findings, a balanced diet (I eat all above) was found to be negatively correlated with HF (OR = 0.194; 95% CI = 0.128–0.294; *P* = 0), CHD (OR = 0.2; 95% CI = 0.133–0.301; meta-*P* < .001), and MI (OR = 0.96; 95% CI = 0.942–0.978; meta-*P* < .001).

Three major cohort studies, the Nurses’ Health Study (NHS, 1980–2012),^[27]^ NHS II (1991–2013),^[28]^ and Health Professionals Follow-Up Study (HPFS, 1986–2012),^[29]^ found that egg consumption was not associated with CVD in US and European populations, but exhibited negative associations with CVD in Asian populations (OR = 0.92, CI: 0.85 to 0.99, *I*^2^ = 44.8%).^[30]^ These cohort studies with longer time spans and large sample sizes strongly support our findings. Our belief is that eggs are rich in beneficial fatty acids like omega-6 and omega-3,^[31]^ and high in protein, all of which have a positive impact on cardiovascular health.^[32,33]^ Milk is a significant biological source of high-quality protein and fatty acids, similar to eggs. Dairy products, in particular, are the main food source of saturated fat and have a higher saturated fat content than nonfat elements.^[34]^ This accounts for approximately one-fifth of the saturated fat intake in the United States.^[35]^ The PURE study, a large-scale epidemiological survey of diet and lifestyle in 21 countries, has confirmed that milk consumption is associated with a reduction in all-cause mortality.^[36]^ Furthermore, extensive research shows that full-fat dairy products play a protective role in preventing CVD,^[36–38]^ bolstering the findings of our study. Additionally, our research suggests that our findings on carbohydrates may be related to energy metabolism processes involving sugar, in addition to dairy and egg products. Disorders of cardiac energy metabolism are known to significantly affect HF. Pathological conditions cause sugar to exhibit a higher efficiency of energy conversion. Manipulating energy substrate utilization from fatty acids to glucose can assist in improving cardiac function and slowing down the progression of HF.^[39]^ Clinical studies have confirmed that sugar supplementation during surgery can reduce MI size, decrease arrhythmia incidence, and shorten overall hospital stays.^[40]^ There is evidence suggesting that low-carbohydrate diets may worsen myocardial damage in diabetic rats by affecting cardiac preload.^[41]^ The Mediterranean diet, which advocates for a nutritionally complete diet, has garnered more attention than diets that focus on enhancing a single aspect. Numerous clinical studies^[42,43]^ have shown the efficacy of the Mediterranean diet in the primary prevention of CVDs. This assertion is widely accepted by scholars. The CORDIOPREV study,^[44]^ conducted on the Mediterranean Diet and CVD in a secondary prevention setting, showed that compared to a low-fat diet, the Mediterranean diet resulted in a greater reduction of major cardiovascular events (crude rate per 1000 person-years: 28.1 [95% CI 27.9–28.3] in the Mediterranean diet group vs 37.7 [37.5–37.9] in the low-fat group, log-rank *P* = .039). These studies are good confirmation of the protective effect of a balanced diet against CVD in our study.

Our study examined the genetic links between dietary habits and CVDs using Genotype Tissue Expression-expression quantitative trait loci MR gene co-localization analysis. Our research indicates that the SNP rs12740374 (PP.H4 = 0.855) had a causal effect on CHD and balanced diet, while the SNP rs7528419 (PP.H4 = 0.716) had a causal effect on MI and balanced diet. It is important to note that these 2 SNPs, rs7528419 and rs12740374, influence shared motifs in 6 genes: KIAA1324, SARS, CELSR2, PSRC1, MYBPHL, and SORT1. PSRC1 (AUC: 0.722), CELSR2 (AUC: 0.657), and MYBPHL (AUC: 0.677) are significant diagnostic markers for CHD and MI. The chromosome 1p13.3 locus, which encodes lipid regulation-related proteins, has been extensively researched in recent years due to its significant implications in CVDs. This locus includes 2 SNPs, rs7528419 and rs12740374, corresponding to the PSRC1, CELSR2, and MYBPHL loci.^[45]^ MYBPHL is a protein that is associated with the structure of myofilaments in atrial tissue. Studies indicate that it may serve as an indicator of atrial damage.^[46]^ Disruptions in MYBPHL function can result in impaired ventricular function and conduction system abnormalities, increasing the risk of arrhythmias and cardiomyopathies in affected individuals.^[47]^ It is worth noting that researchers have increasingly recognized rs12740374 as a significant risk locus for various CVDs, including coronary artery disease, heart valve disease, peripheral vascular disease, and ischemic stroke.^[48–50]^ The variants of this locus may regulate the CELSR2 and SORT1 genes, potentially affecting liver-specific regulation and the production of low-density cholesterol.^[51]^ More precisely, research suggests that mutations in the SORT1 gene at rs12740374, located at locus 1p13.3, lead to impaired hepatic regulation of plasma LDL-C and VLDL particle levels.^[52]^ Additionally, genetic polymorphisms in PSRC1, CELSR2, and SORT1 based on the rs2740374 mutation may be linked to dose variability in warfarin use for CVD.^[53]^ Similar to rs12740374, research on rs7528419 has focused on its association with CHD and lipid metabolism. Studies have shown that the rs7528419-G allele of the CELSR2 gene provides protection against CHD in men.^[54]^ Additionally, there may be a connection between variation in rs75,28,419, found in the SORT1 locus (specifically liver), and progranulin and lipoprotein-associated phospholipase A(2) metabolism.^[54,55]^

This study is the first to comprehensively examine the effects of a diet that includes carbohydrates, eggs, and dairy products on various CVDs. The findings suggest that Never eat eggs, dairy products, or sugar is a significant risk factor for CVD in all groups, while a balanced diet can offer protection against most CVDs. Further exploration and investigation of rs12740374 and rs7528419, along with their corresponding genes PSRC1, CELSR2, and MYBPHL, are recommended as potential pharmacological targets for the treatment of CHD and MI. The study provides a theoretical foundation for the impact of carbohydrates, egg products, and dairy products on CVD. Additionally, it aims to offer more comprehensive data and guidance on planning the dietary structure for patients with CVD in the clinic. However, this study has some limitations. We did not separate various diet varieties, and we did not conduct a positive control study on over-diet. Our current research on PSRC1, CELSR2, and MYBPHL genes is preliminary. Our future direction will focus on further investigation. We encourage researchers to pay more attention to the impact of dietary structure on CVD.

## Author contributions

**Conceptualization:** Youfu He, Qiang Wu, Yu Qian.

**Data curation:** Youfu He, Qiang Wu, Yu Qian.

**Formal analysis:** Youfu He, Qiang Wu, Yu Qian.

**Funding acquisition:** Youfu He, Qiang Wu.

**Investigation:** Youfu He, Qiang Wu, Debin Liu.

**Methodology:** Youfu He, Debin Liu.

**Project administration:** Youfu He, Debin Liu.

**Resources:** Youfu He.

**Software:** Youfu He, Debin Liu, Xuantong Meng, Yu Qian.

**Supervision:** Youfu He, Xuantong Meng, Yu Qian.

**Validation:** Youfu He, Xuantong Meng, Yu Qian.

**Visualization:** Youfu He.

**Writing – original draft:** Youfu He.

**Writing – review & editing:** Youfu He.

## Supplementary Material


