# Supplementary material for: Effect of dietary habits on multiple cardiovascular diseases: A comprehensive Mendelian randomization study
Source: Medicine (Baltimore). 2025 Sep 5;104(36):e44352. doi: 10.1097/MD.0000000000044352 (PMC12419428; doi:10.1097/MD.0000000000044352)
Supplement: Supplementary file 2 [file medi-104-e44352-s002.docx]

**Supplementary Figure S1. Plot of results of MR analysis of dietary habits and HF.**


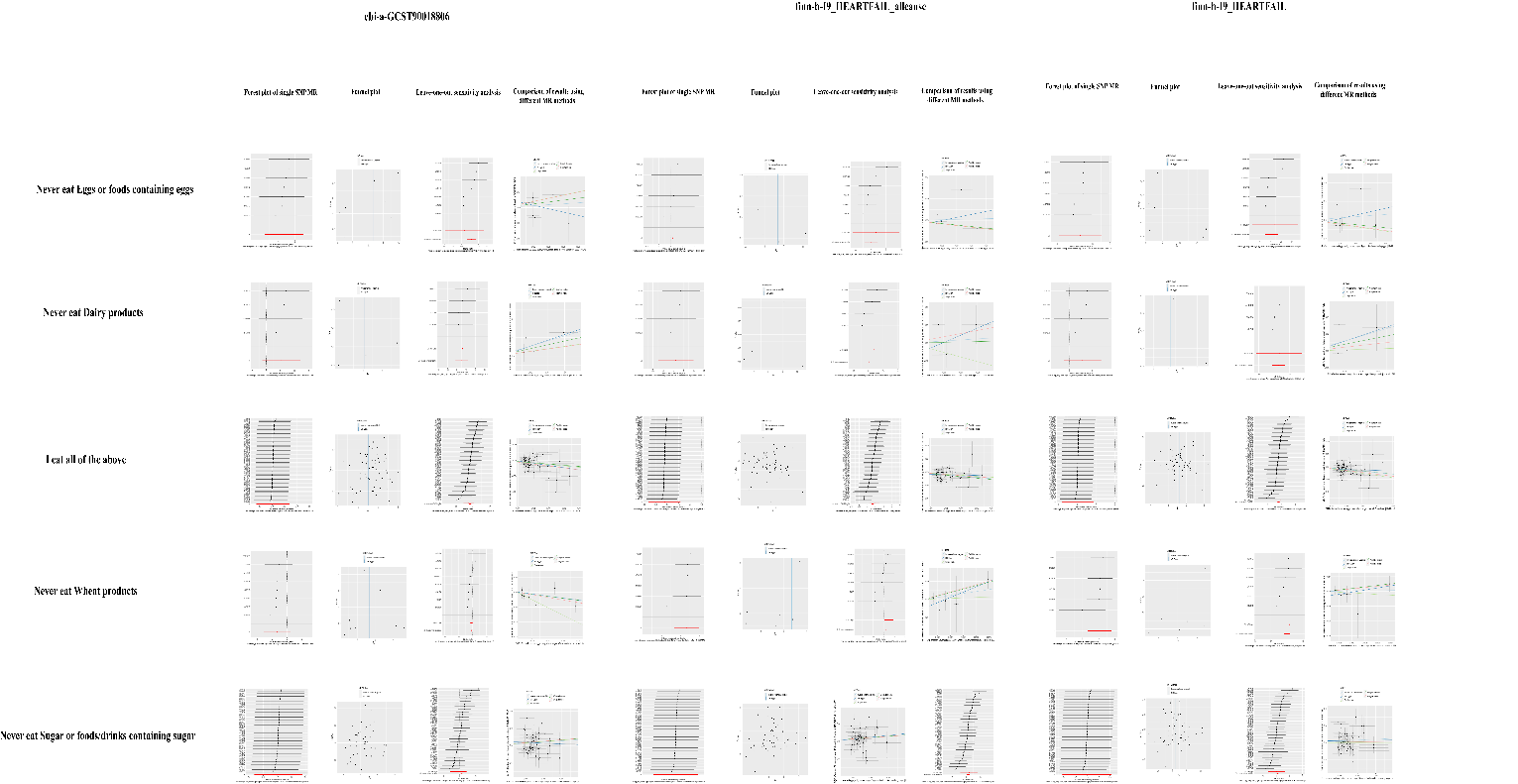


**Supplementary Figure S2. Plot of results of MR analysis of dietary habits and essential hypertension.**


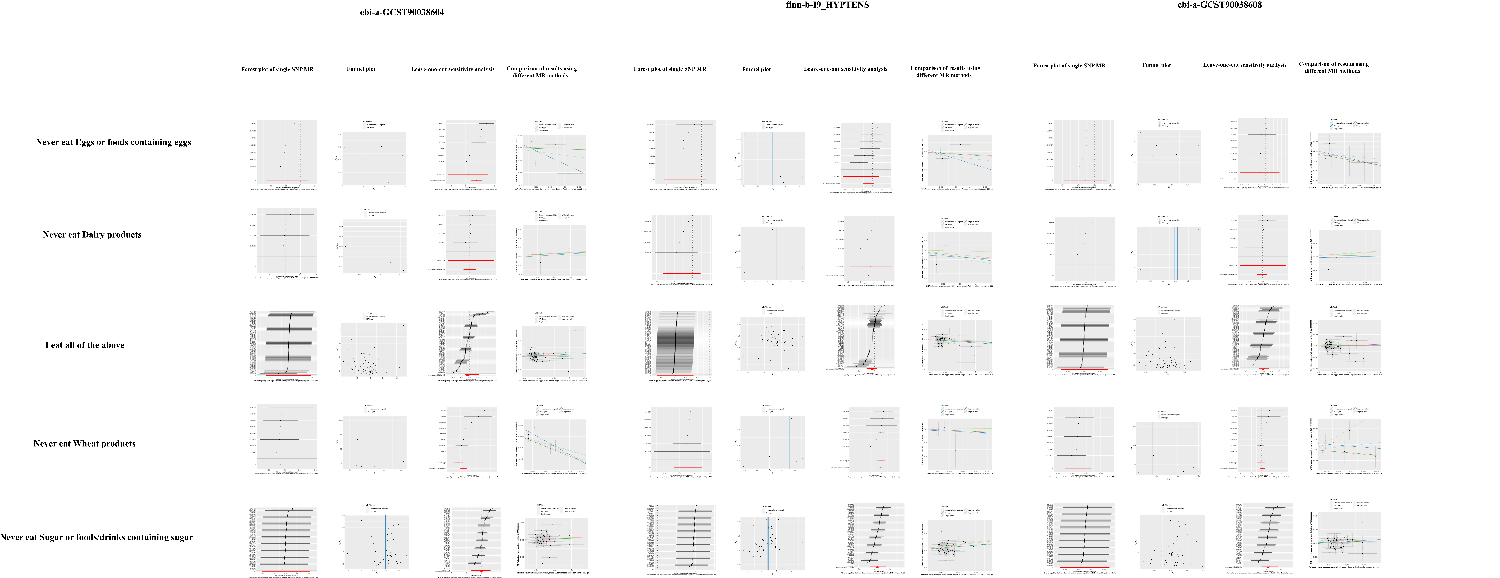


**Supplementary Figure S3. Plot of results of MR analysis of dietary habits and CHD.**

**
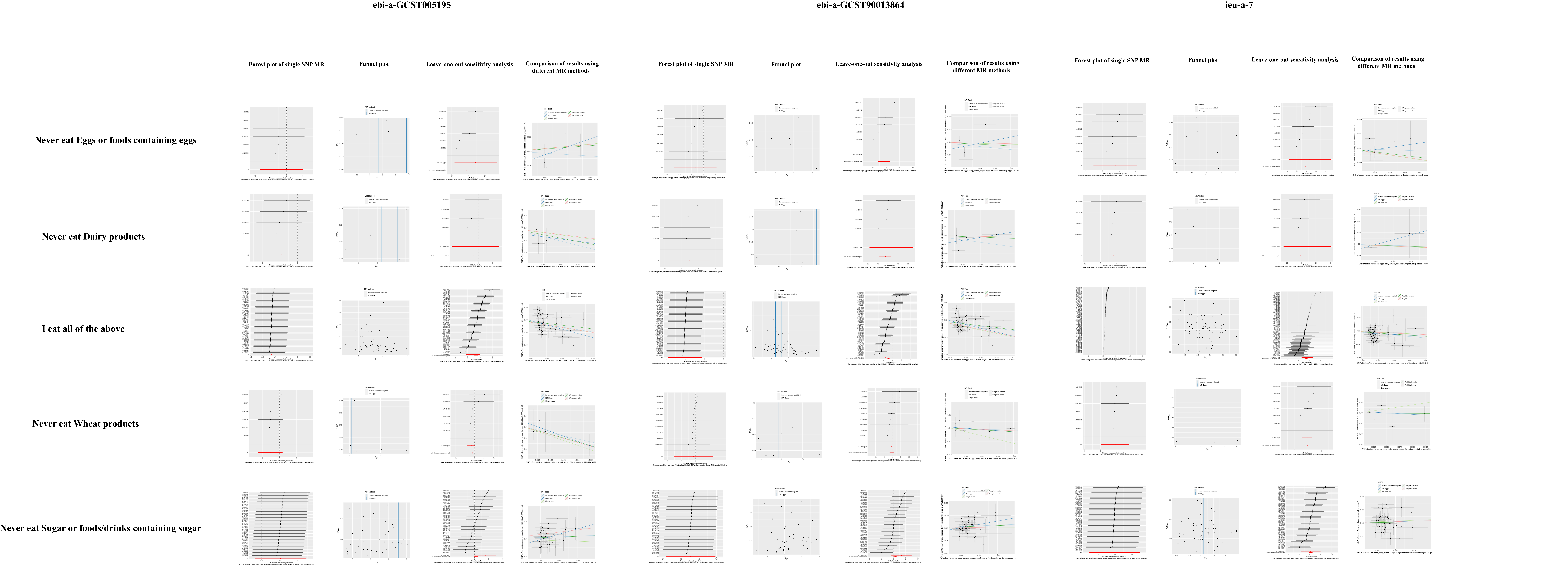
**

**Supplementary Figure S4. Plot of results of MR analysis of dietary habits and MI.**

**
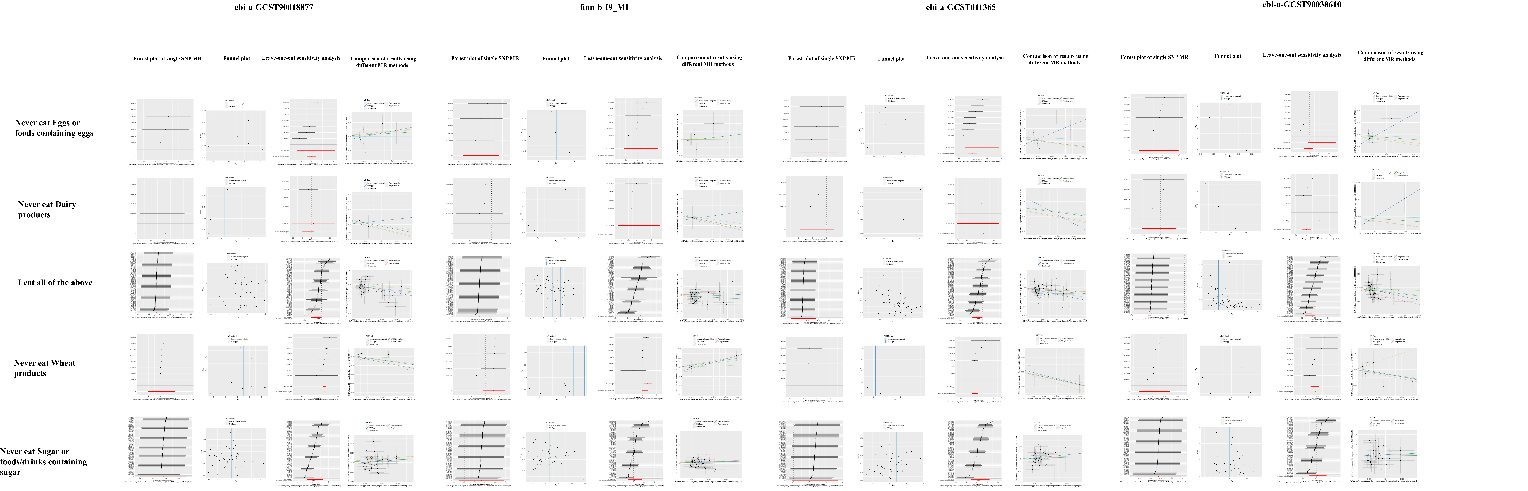
**

**Supplementary Figure S5. Plot of results of MR analysis of dietary habits and myocarditis.**

**
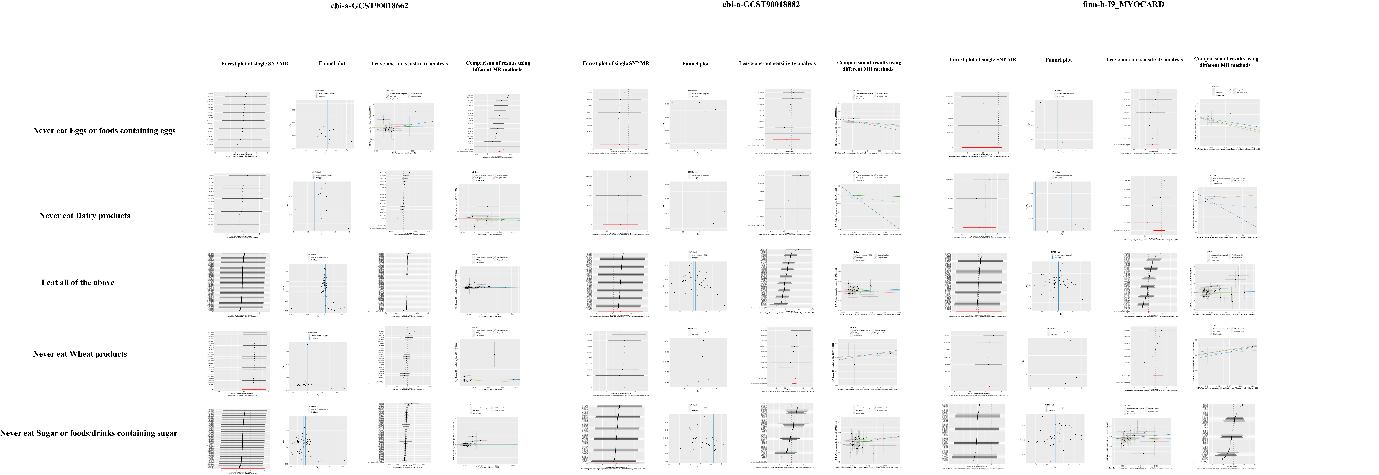
**

**Supplementary Figure S6. Plot of results of MR analysis of dietary habits and cardiomyopathy.**

**
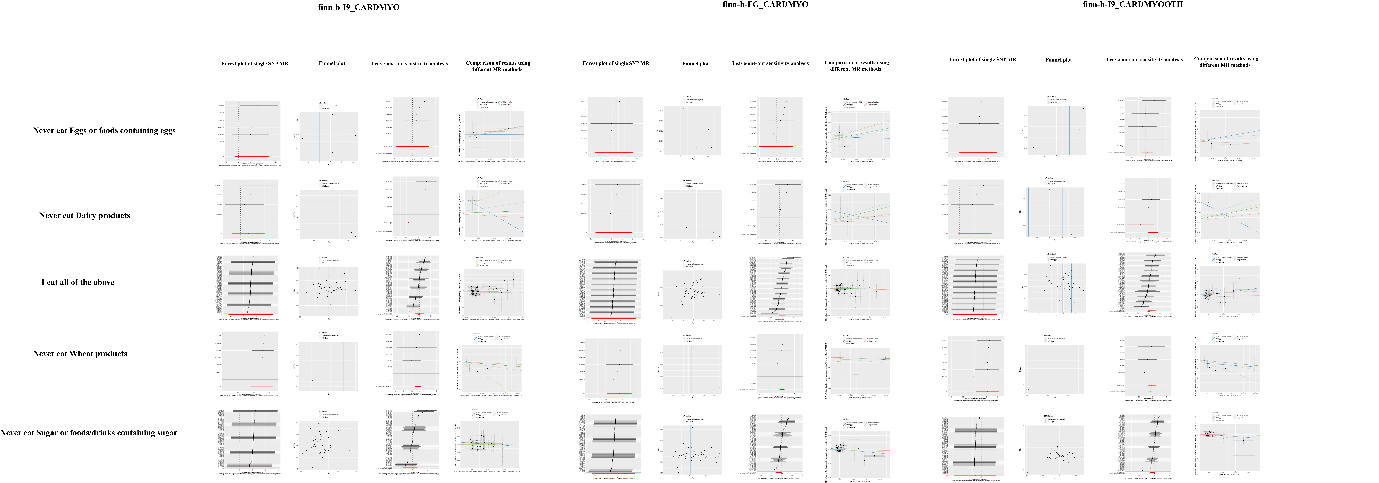
**

**Supplementary Figure S7. Plot of results of MR analysis of dietary habits and arrhythmia.**

**
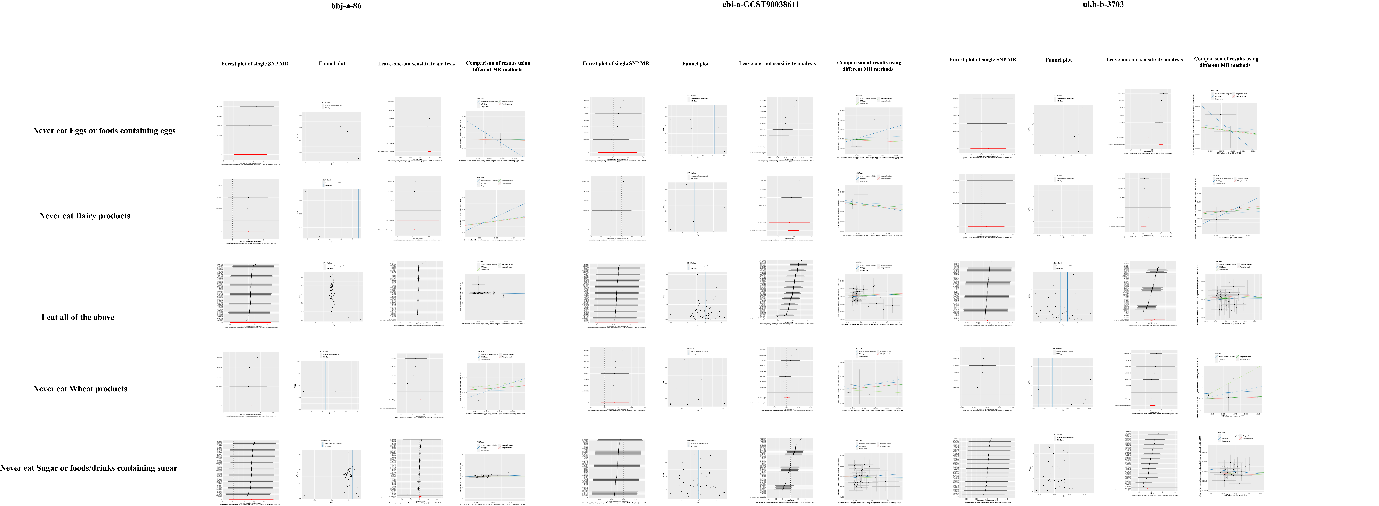
**

**Supplementary Figure S8. Plot of results of MR analysis of dietary habits and valvulopathy.**

**
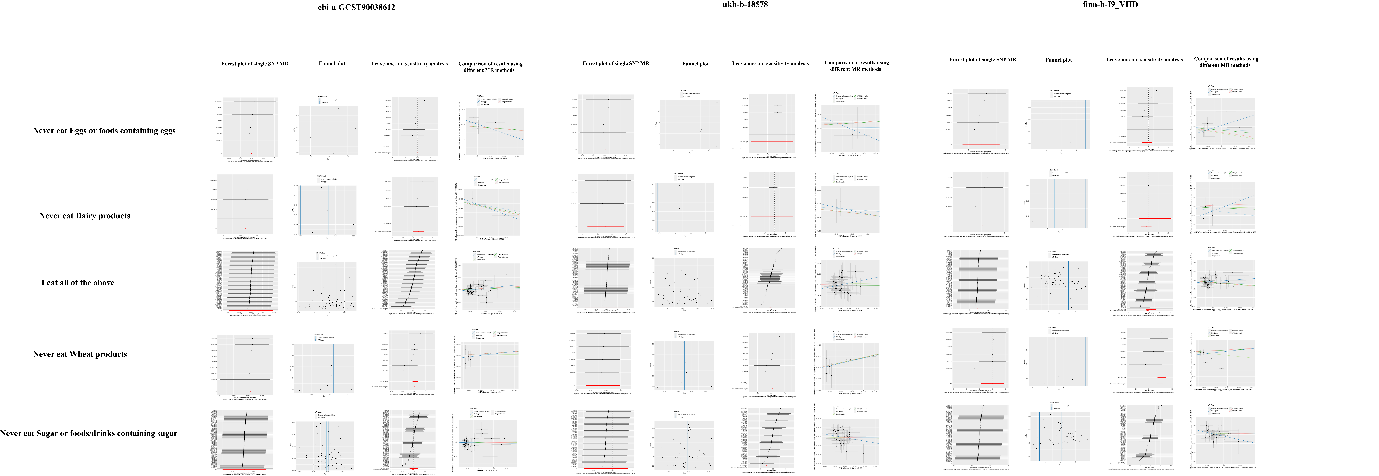
**

**Supplementary Figure S9. Plot of results of MR analysis of dietary habits and cardiac death.**

**
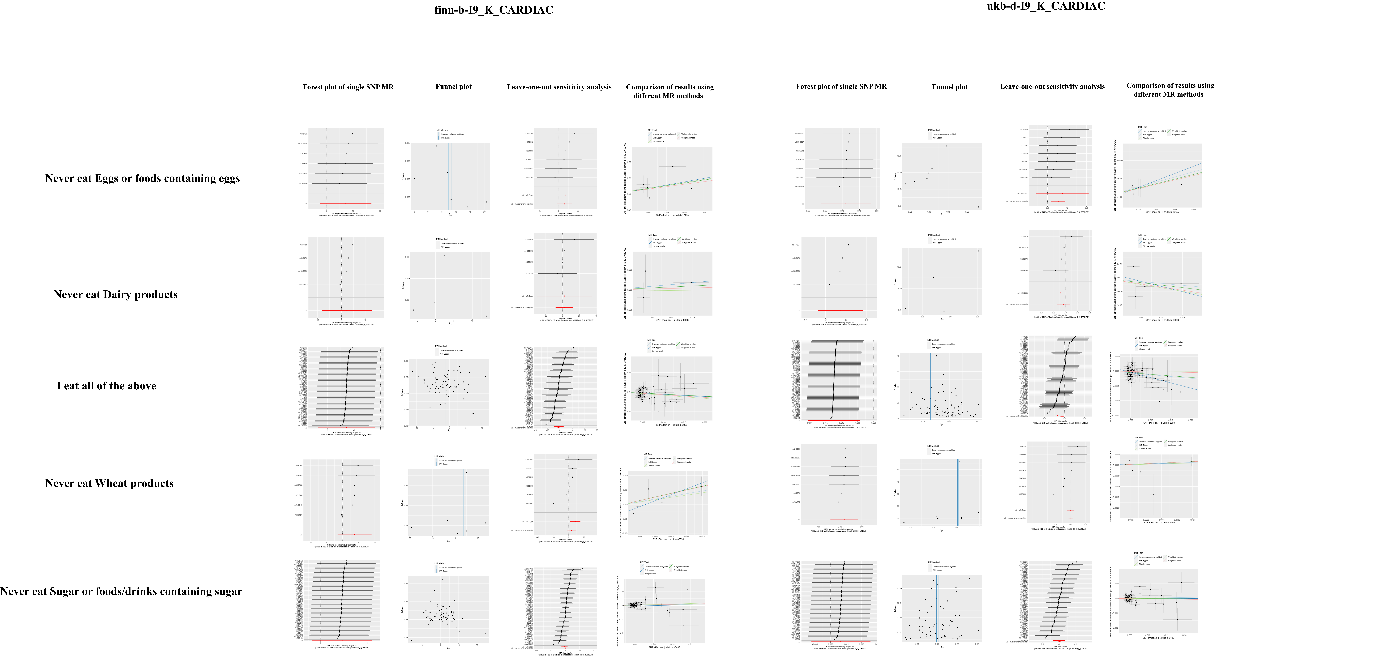
**

**Supplementary Figure S10. Forest plot of different dietary habits and results of MR analysis of cardiovascular diseases.**

**
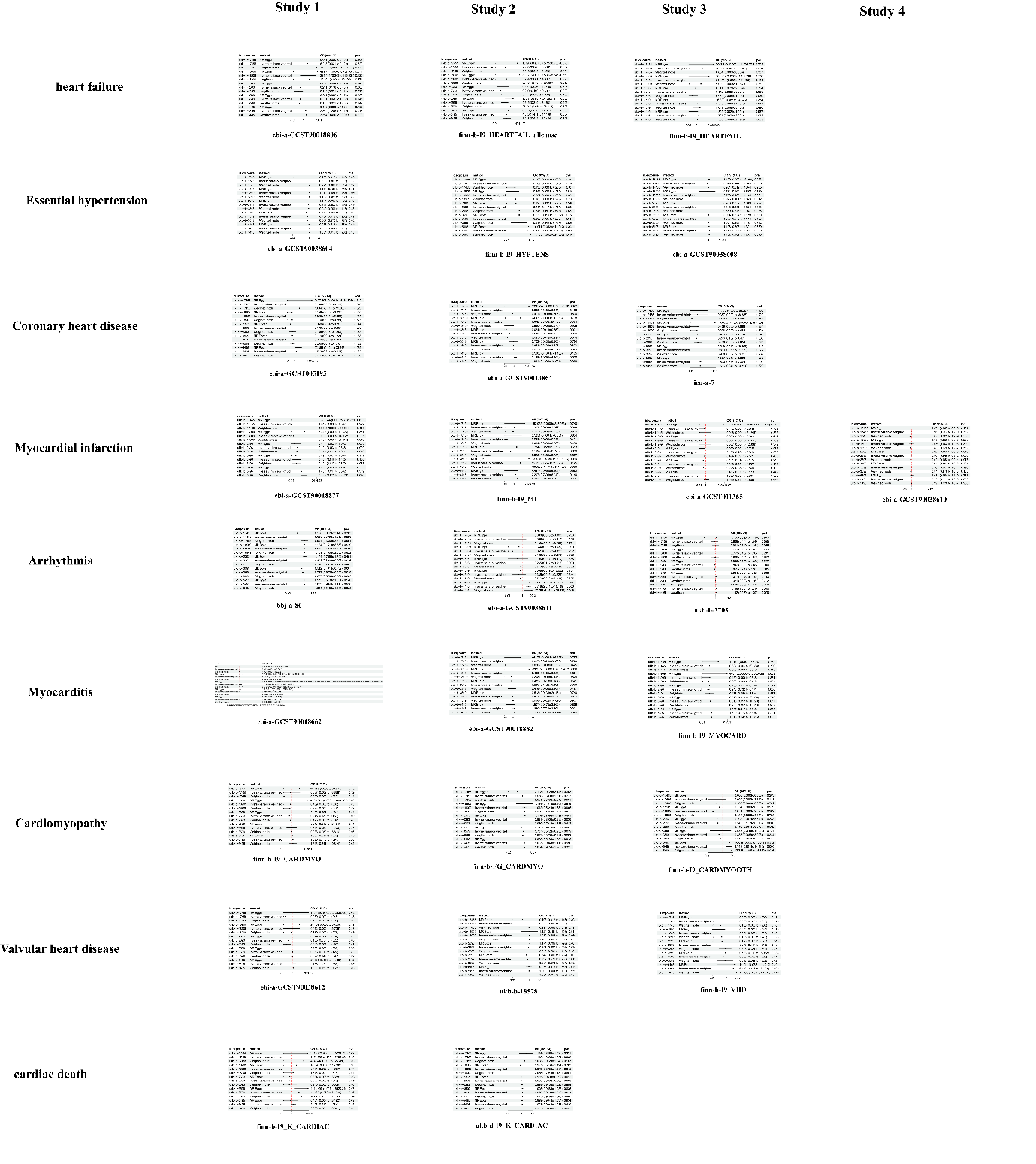
**

**Supplementary Figure S11: Results of meta-analysis.**

**
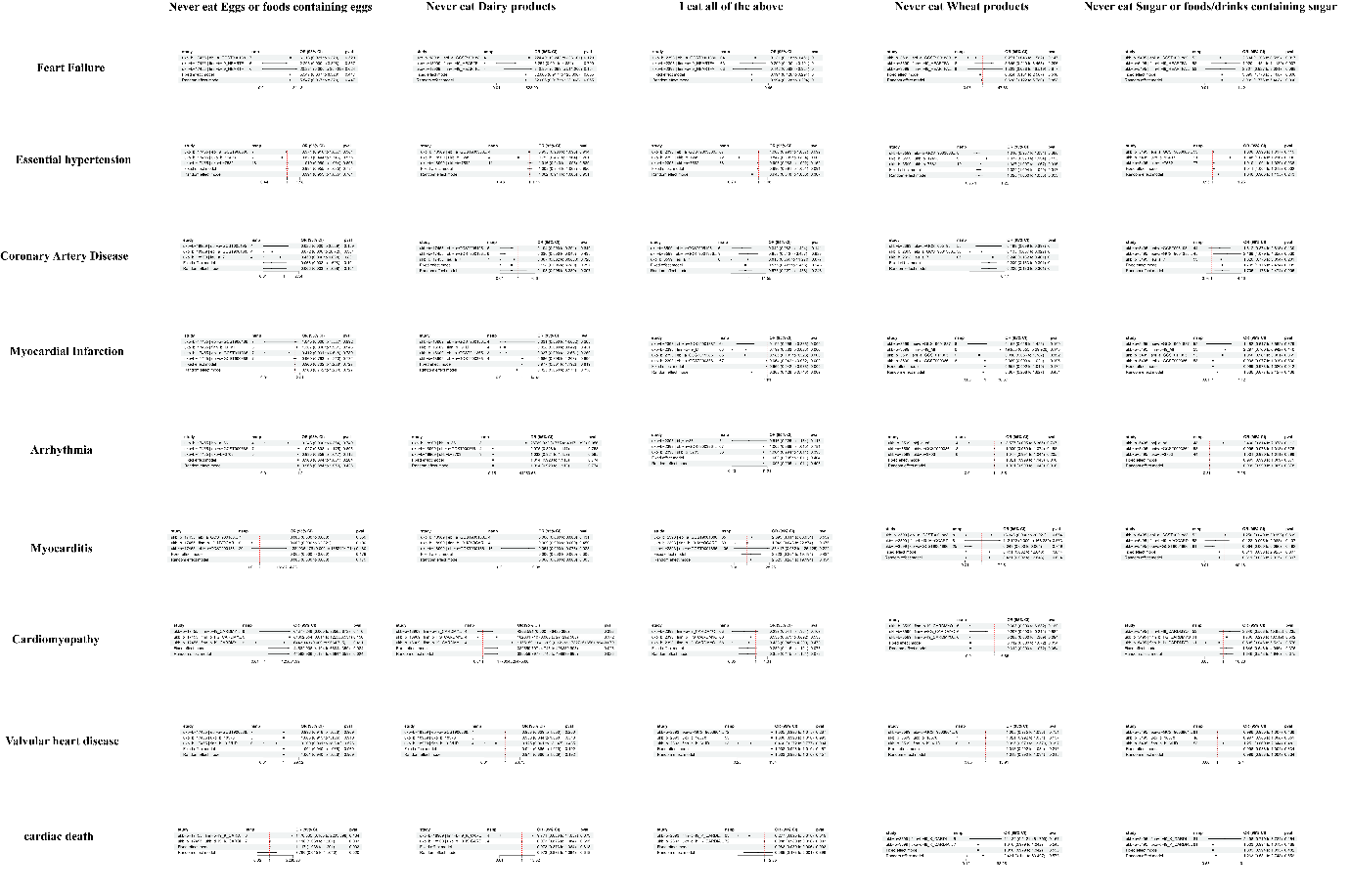
**
